# Nanocatalytic NO gas therapy against orthotopic oral squamous cell carcinoma by single iron atomic nanocatalysts

**DOI:** 10.1080/14686996.2024.2368452

**Published:** 2024-06-28

**Authors:** Yuting Xie, Jiaxin Zuo, Angang Ding, Ping Xiong

**Affiliations:** Department of Ultrasound, Shanghai Ninth People’s Hospital, Shanghai Jiaotong University School of Medicine, Shanghai, P. R. China

**Keywords:** Single-atom catalyst, nitric oxide, nanocatalytic therapy, oral squamous cell carcinoma therapy

## Abstract

Oral squamous cell carcinoma (OSCC) has been being one of the most malignant carcinomas featuring high metastatic and recurrence rates. The current OSCC treatment modalities in clinics severely deteriorate the quality of life of patients due to the impaired oral and maxillofacial functions. In the present work, we have engineered the single-atom Fe nanocatalysts (SAF NCs) with a NO donor (S-nitrosothiol, SNO) via surface modification to achieve synergistic nanocatalytic NO gas therapy against orthotopic OSCC. Upon near-infrared laser irradiation, the photonic hyperthermia could effectively augment the heterogeneous Fenton catalytic activity, meanwhile trigger the thermal decomposition of the engineered NO donor, thus producing toxic hydroxyl radicals (•OH) and antitumor therapeutic NO gas at tumor lesion simultaneously, and consequently inducing the apoptotic cell death of tumors via mitochondrial apoptosis pathway. This therapeutic paradigm presents an effective local OSCC therapeutics in a synergistic manner based on the nanocatalytic NO gas therapy, providing a promising antitumor modality with high biocompatibility.

## IMPACT STATEMENT

In this work, we have engineered the NIR-triggered NO liberating donor module RSNO onto single-atom Fe nanocatalysts for synergized nanocatalytic therapy and NO gas therapy against orthotopic OSCC with high therapeutic selectivity, efficacy and biocompatibility.

## GRAPHICAL ABSTRACT

**Figure:**
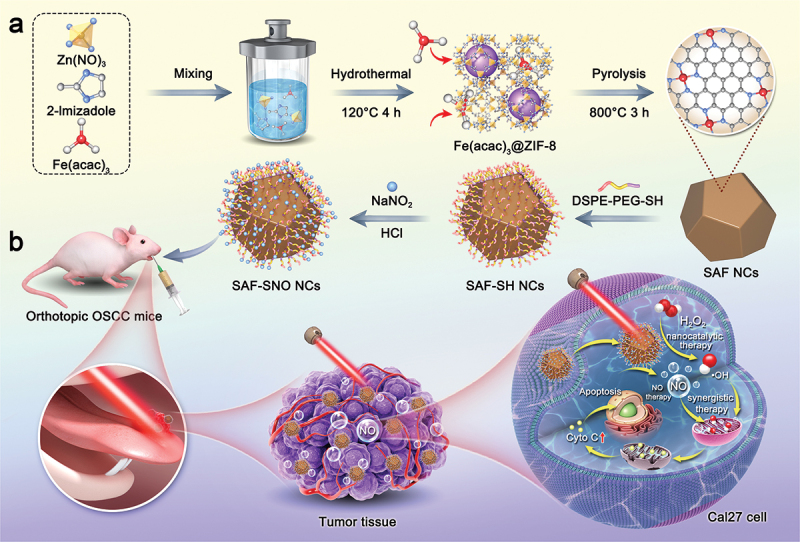

## 1. Introduction

Oral squamous cell carcinoma (OSCC) is one of the most common type of head and neck squamous cell carcinoma (HNSCC) worldwide [1–3]. With its high malignancy, metastasis and recurrence, the overall 5-year survival rate of OSCC patients is typically below 50% [4,5]. Surgical resection combined with chemotherapy and radiation have been the predominant treatment modalities against OSCC in many cases [6–8]. However, postoperative patients may experience severe impairment of physiological functions including speech and eating [9,10]. Meanwhile, radiotherapy and chemotherapy bring terrible side effects such as mucositis, salivary gland dysfunction, and hematological toxicity to the patients [11]. Considering these drawbacks, establishment of non-invasive or minimally invasive alternative therapeutic strategy against OSCC is emerging to meet the clinical demand.

Heterogeneous Fenton catalysis enabled by single atom iron nanocatalysts have been the most promising and emerging catalytic modality in triggering prominent oxidative stress inside tumor region [12,13], in responsive to the key hallmarks (lactic acidosis and intracellular accumulation of peroxides) of the tumor microenvironment (TME) [14,15]. During the heterogeneous Fenton catalytic process, the single atom iron sites [16,17] within the nanocatalysts could effectively catalyze the intracellular hydrogen peroxide for highly toxic hydroxyl radicals (•OH) generation [18] via heterogeneous Fenton-like reaction [19,20], establishing the profound basis for tumor catalytic destruction [21,22].

Nitric oxide (NO) is a dominant gas signaling molecule regulating a series of physiological and pathological processes including apoptosis, angiogenesis, and immune response [23–25]. It has been demonstrated that NO play dual modulation roles within tumor cells [26]. At low plasma NO concentration, it could specifically promote the growth of the tumor cells. While at higher concentration (>1 μM), antitumor effect could be observed, depending on the site and liberation rates [27]. Introduction of NO has been the most attractive and effective strategy for antitumor modality. However, short plasma half-life lack of the on-demand release property of NO may decrease the accumulation amount of NO at specific lesion sites, subsequently limiting the therapeutic efficacy. Therefore, on-demand trigger-release of NO gas within the tumor tissue is of critical significance. Recently, many efforts have been devoted to the development of stimuli-responsive nanomaterials acting as NO donors to trigger local NO release in a controlled manner, such as bis-N-nitroso compounds (BNNs) [28], S-nitrosothiols (RSNOs) [29] and diazeniumdiolates (NONOates) [30]. Compared to other NO donors, RSNO feature with unique advantage of high biocompatibility and the on-demand trigger-release performance [31]. RSNO can serve as the NO liberator upon certain endogenous and external stimulus including pH changes, enzymes, transition metal ions, light, electricity and magnetism [30,32]. Among these stimulus, near-infrared irradiation (NIR) has the most dominant controllability to accurately trigger tissue hyperthermia and the responsiveness with well penetration depth and minimally invasive characteristic. RSNO could be easily cleaved for NO liberation upon NIR, synergizing the therapeutics with high effectiveness.

**Scheme 1.**
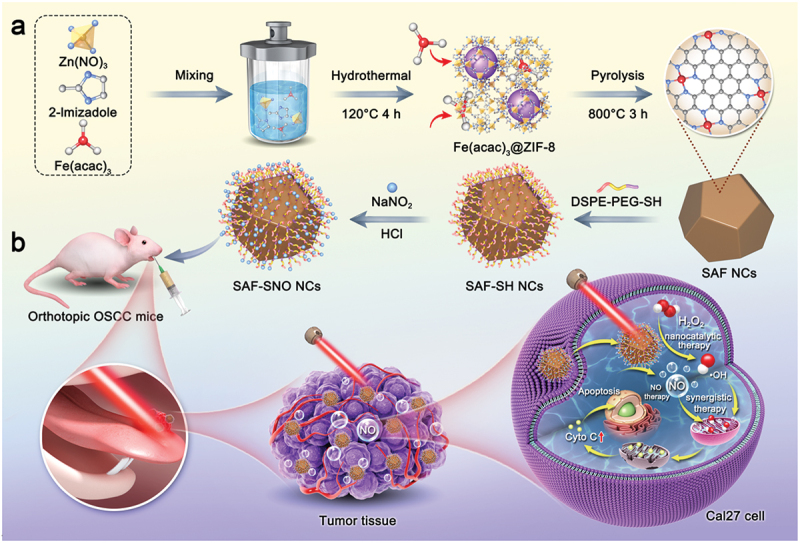
Schematic illustration of (a) SAF-SNO NCs synthesis and (b) synergistic therapy of SAF-SNO NCs against orthotopic OSCC.

In this work, we have engineered the NIR-triggered NO liberating donor module RSNO onto single-atom Fe nanocatalysts (SAF NCs) through surface modification, constructing the S-nitrosothiols functionalized SAF NCs (SAF-SNO NCs) (Scheme 1). Upon NIR laser irradiation, the as-synthetic SAF-SNO NCs not only exhibit augmented heterogeneous Fenton catalytic activities in generating abundant cytotoxic hydroxyl radicals, but also trigger the controllable and sustained NO gas production for antitumor therapeutics. Considering the urgent therapeutic demands for OSCC, we adopted intratumoral administration of SAF-SNO NCs in treatment of the primary OSCC, which promises the efficient accumulation of SAF-SNO NCs at lesion site of tongue without major side-effects. The present therapeutic paradigm integrates the nanocatalytic heterogeneous Fenton reactions with NO gas therapeutics for local treatment of primary OSCC, revealing promising clinical translation perspectives for effective oral cancer therapeutics with high biocompatibility.

## 2. Material and methods

### 2.1. Material and reagents

Zinc nitrate hexahydrate (Zn(NO_3_)·6 H_2_O, 99.99%), hydrochloric acid (HCl, 36.0~38.0%) and sodium nitrite (NaNO_2_, 99.0%) were purchased from Sinopharm Chemical Reagent Co., China. Iron (III) acetylacetone (Fe(acac)_3_) was obtained from Sigma-Aldrich Co., US and 2-methyimidazole (99%) was obtained from J&K Co., China. 1,2-distearoyl-sn-glycero-3-phosphoethanolamine-N-[thiol (polyethylene glycol)] (DSPE-PEG-SH) was purchased from Shanghai Ponsure Biotech, Inc.

### 2.2. Characterizations

Transmission electron microscopy (TEM) images, energy-dispersive X-ray spectroscopy (EDS) and corresponding EDS-maps were acquired with a JEM-2100F microscopy (JEOF, Japan) operated at 200 kV. The Zetasizer Nanoseries (Malvern, UK) was applied to dynamic light scattering analysis to decide the hydrodynamic sizes and zeta potential. X-ray photoelectron spectroscopy (XPS) was applied on an ESCAlab250 (Thermal Scientific, US). Fourier transform infrared spectroscopy (FTIR, Thermo Scientific, US) was used to analyze the chemical bonds of nanoparticles. The thermal-field video and corresponding temperature monitoring were recorded by an infrared thermal imaging instrument (FLIR A325SC camera, US). The confocal laser scanning microscopy (CLSM) images were acquired on FV1000 microscope (Olympus Company, Japan). Cell apoptosis analysis were captured by a BD LSRFortessa flow cytometry (BD, US). NIR laser irradiation was generated by an 808 nm high-power multimode pump laser (Shanghai Connect Fiber Optics Company, China).

### 2.3. Synthesis of SAF nanocatalysts

The SAF NCs were fabricated by an ‘isolation-pyrolysis’ method [21]. Zn(NO_3_)·6 H_2_O (4 mmol) and Fe(acac)_3_ (2.5 mmol) were dispersed totally in methanol (30 mL) by violent sonication. 2-Methylimidazole (16 mmol) was dissolved in methanol (15 mL) under mild stirring. Then mixing of the solution drop by drop to form ZIF-8 nanoparticles (NPs). After 1 h reaction, the product was then placed in a stainless-steel autoclave with a Teflon liner, sealed and heated to 120°C in an oven for 4 h. After centrifugation and washed with dimethylformamide (DMF) and methanol three times, the Fe(acac)_3_@ZIF-8 NPs were produced. The light orange powder obtained by cold drying was pyrolyzed under flowing argon protection at a gentle heating rate of 2°C min^−1^ for 3 h at 800°C. The SAF NCs containing single iron sites were collected after cooling.

### 2.4. Surface modification and NO donor conjugation of SAF-SH nanocatalysts

To graft S-nitrosothiols (R-SNO) group into SAF NCs, the nanocatalysts initially modified with -SH group and improve the hydrophilicity. SAF NCs (0.02 g) were diluted in DMF (30 mL) upon violent sonication for 30 min at room temperature (RT) and then put into a round-bottom flask. DSPE-PEG-SH (0.04) gwas also dispersed in DMF (5 mL) with stirring to dissolved fully into a homogeneous solution. Followed by dropwise addition into SAF aqueous solution, and stirred for 24 h protecting from light at RT. After washing with DMF, ethanol and deionized water for 3 times, respectively, SAF-SH NCs were collected and uniformly dispersed in deionized water. Then, followed dropwise adding NaNO_2_ solution(6 μM)and diluted HCl (30 μM) into SAF-SH solution (deionized water, 10 mL) and stirring for 2 h in the dark environment with the condition of low temperature, the resulting SAF-SNO NCs products were acquired by centrifugation and washed with deionized water for 3 times.

### 2.5. In vitro hydroxyl radical evaluation s of SAF-SNO nanocatalysts

3,3’,5,5’-Tetramethyl benzidine (TMB) was adopted to explore the hydroxyl radicals (•OH) production capacity. SAF-SNO NCs (25 µg mL^−1^) and TMB (0.8 mg ml^−1^) were added into a phosphate-buffered solution with the pH value of 6.8 containing vary concentrations of H_2_O_2_ (0, 12.5, 25, 50 and 100 µM), then the characteristic absorption peak located at 650 nm was recorded. In addition, vary doses of SAF-SNO NCs (12.5, 25, 50 and 100 µg mL^−1^) reacting with TMB (0.8 mg ml^−1^) at the same concentration of H_2_O_2_ (100 µM) were also detected. For the electron spin resonance (ESR) experiment, 5,5-dimethyl-1-pyrroline N-oxide (DMPO) was applied as the trap for •OH. Typically, DMPO (25 mM) was added into the buffer system (pH = 7.4 or 6.8) containing H_2_O_2_ (100 µM) and SAF-SNO NCs (1 mg mL^−1^, 50 µL). After sufficient reaction, the solution was transferred to a quartz tube for ESR assay on Bruker EMX1598 at RT.

### 2.6. In vitro photothermal performance of SAF-SNO nanocatalysts

To assess the photothermal performance of SAF-SNO solution, the infrared thermography was used to record temperature variations during 808 nm laser irradiation. To record temperature-changes curves, various concentrations of SAF-SNO aqueous solutions (0, 20, 40, 80 and 100 µg mL^−1^) were irradiated by 808 nm laser at 2 W cm^−2^ for 8 min. The temperature of SAF-SNO aqueous solutions (100 µg mL^−1^) under NIR laser irradiation at different power densities (0, 0.5, 1.0, 1.5, and 2 W cm^−2^) for 5 min also measured. Therefore, the extinction coefficient and photothermal-conversion efficiency of SAF-SNO were calculated. Subsequently, temperature changes of SAF-SNO solution (100 µg mL^−1^) through five laser on/off cycles (1.5 W cm^−2^) were recorded to evaluate the photothermal stability.

### 2.7. Measurement of NO release

The NO generation from SAF-SNO was quantitatively assessed by a Griess assay. After various treatments, including different irradiation power densities (0, 0.5, 1.0, 1.5 and 2.0 W cm^−2^) and varied concentrations (0, 6.25, 12.5, 25, 50, 100 and 150 µg mL^−1^), the Griess agent was added into different groups and recorded by a microplate reader to evaluate the release of NO from SAF-SNO.

### 2.8. CLSM analysis observation of intracellular endocytosis

Human tongue squamous cancer cells (Cal27 cells) were seeded into CLSM-specific culture dishes at a density of 1 × 10^5^ and incubated overnight at 37°C, after which the medium was replaced by SAF-SNO conjugated with fluorescein isothiocyanate (FITC) (1 mL, 100 µg mL^−1^) and then co-cultured for several hours. Cell nucleus was stained with DAPI for 20 min after washing the medium with phosphate-buffered saline (PBS) three times and then CLSM images were recorded.

### 2.9. In vitro cytotoxicity assay

Cal27 cells were incubated in Dulbecco’s modified Eagle’s medium (DMEM, high glucose, GIBCO) with 10% fetal bovine serum (FBS) and 1% penicillin/streptomycin under 5% CO_2_ at 37°C. The cells were seeded at a density of 1 × 10^4^ cells/well in 96-well culture plates for 24 h. Neutral or acidic fresh DMEM medium (pH adjusted by diluted HCl) containing H_2_O_2_ (100 µM), SAF-SH NCs or SAF-SNO NCs ([Nb] = 0, 25, 50, 100, 200 and 400 µg mL^−1^) were replaced for further co-incubation. After rinsing with PBS, cell viability was performed using cell-counting kit-8 (CCK-8) according to the protocols.

### 2.10. In vitro synergistic therapy performance of SAF-SNO against Cal27 cells

Cal27 cells were seeded into CLSM-specific culture dishes at a density of 1 × 10^5^ and incubated overnight at 37°C, after which the medium was replaced by FITC-conjugated SAF-SNO (1 mL, 100 µg mL^−1^) and then co-cultured for several hours. Cell nucleus was stained with DAPI for 20 min after washing the medium with PBS three times and then CLSM imaging was observed on an Olympus FV1000 laser scanning microscope.

Cal27 cells were seeded in 96-well plates at a density of 1 × 10^4^ cells/well for 12 h to attach on the plates, then co-incubated with SAF-SH and SAF-SNO NCs at varied concentrations (0, 25, 50, 100, 200 µg mL^−1^) for 6 h to allow endocytosis. Following, these cells were taken a standard CCK-8 protocol when exposed to 808 nm laser irradiation for 5 min at 1.5 W cm^−2^. In addition, the cells viability was also evaluated after irradiation at different power densities (0, 0.5, 1.0, 1.5, and 2.0 W cm^−2^). Synergistic therapies effect of SAF-SNO NCs was also observed by CLSM using Calcein/PI Cell Viability/Cytotoxicity Assay Kit.

Cal27 cells were pre-seeded into CLSM-specific dishes at a density of 1 × 10^5^ and cultured for 12 h to attach on the plates. Then, the cells were treated with PBS, SAF-SH, SAF-SNO (100 µg mL^−1^) and H_2_O_2_ (100 µM) for 6 h. Then, these cells were irradiated for 5 min using 808 nm laser at different power densities (0 and 1.5 W cm^−2^). After treatments, Cal27 cells were observed by CLSM, in which live cells and dead cells were stained by Calcein-AM and PI, respectively.

Flow cytometry (Annexin V-FITC apoptosis detection kit) was applied to quantitatively measure cell apoptosis levels. Typically, Cal27 cells were seeded into 6-well plates for 12 h to stick to the dishes, then the medium was added by PBS, SAF-SH, SAF-SNO (100 µg mL^−1^) and H_2_O_2_ (100 µM) incubated for 8 h. Following irradiated for 5 min using 808 nm laser, the cells were collected by using trypsin, centrifugation and washing for 3 times with PBS. Finally, 5 µL PI and 10 µL FITC were added into these cells for 20 min incubation against light. The flow cytometry was then used to evaluate cell-apoptosis levels.

### 2.11. Intracellular free radical and NO production

For cell staining, 1 × 10^5^ Cal27 cells suspended in DMEM medium (1 mL) was inoculated into the confocal-specific culture disk. Cells were co-incubated with SAF-SNO NCs (100 µg mL^−1^) and H_2_O_2_ (100 µM) under natural or acidic medium for 12 h. Then the •OH-sensitive probe 2′,7′-dichlorofluorescin diacetate (DCFH-DA, 10 μM) was employed to the confocal observation. Additionally, the fluorogenic probe, 3-Amino,4-aminomethyl-2’,7’-difluorescein diacetate (DAF-FM DA), was applied to verify the intracellular NO release. After exposed to 808 nm laser at different power densities (0, 0.5, 1, 1.5, 2.0 W cm^−2^), Cal27 cells were incubated with DAF-FM DA (5 µM) for 20 min. The CLSM images of released NO were captured by excitation at 488 nm.

Furthermore, to understand the mechanism of tumor growth inhibition, the mitochondrial membrane potential was detected by 5,5′,6,6′-tetrachloro-1,1′,3,3′-tetraethylbenzimidazoly carbocyanine iodide (JC-1, Beyotime Biotechnology). After incubated with SAF-SNO NCs (100 µg mL^−1^) and H_2_O_2_ (100 µM) under natural or acidic medium for 4 h and exposed to 808 nm laser for 5 min, Cal27 cells were loaded with JC-1 dye (2 μM) and incubated for 20 min at 37°C, followed washing with 1 × PBS for three times before flow cytometry analysis. Then, the apoptotic proteins (p53, Bax, Cyt c, Caspase-3, Caspase-9) expression levels were tested by western blotting. After different treatments, proteins were then extracted with RIPA buffer (50 mM Tris HCl pH = 8, 150 mM NaCl, 1% NP-40 and 0.5% sodium deoxycholate) and separated by 12% native PAGE in the absence of sodium dodecyl sulfate (SDS). The decomposed proteins were transferred to polyvinylidene difluoride (PVDF) membranes, which and were then incubated overnight at 4°C with the antibody against p53, Bax, Cyt c, Caspase-3 and Caspase-9 (Cell Signaling Technology, U.S.A.). Thereafter, the membranes were incubated with anti-rabbit horseradish peroxidase (HRP)-labelled secondary antibody solution for 2 h at room temperature. Western Lightning Plus-ECL reagent (Perkin Elmer) was added for band visibility.

### 2.12. In vivo toxicity assay

The Animal experiment procedures were confirmed to the guidelines for the Animal Care Ethics Commission of Shanghai Ninth People’s Hospital, Shanghai Jiaotong University School of Medicine (SH9H-2019-A176-1). Twenty 6-week-old healthy female BALB/c nude mice (~20 g) were randomly divided into four groups (*n* = 5) and then intravenously administered with PBS, SAF-SH, SAF-SNO (20 mg kg^−1^) via tail veins, which then fed for 14 days to evaluate *in vivo* biotoxicity. The blood samples were collected for serum biochemistry assays and complete blood panel tests, including red blood cells (RBC), white blood cells (WBC), platelets (PLT), mean corpuscular volume (MCV), hemoglobin (HGB), mean corpuscular hemoglobin (MCH), mean corpuscular hemoglobin concentration (MCHC), and albumin (ALB), total protein (TP), globulin (GLB), blood urea nitrogen (BUN), creatinine (CREA), total bilirubin (TBIL), alanine transaminase (ALT), and aspartate transaminase (AST). The major organs (heart, liver, spleen, lung and kidney) were removed at last day, fixed in 10% paraformaldehyde, sectioned, mounted on glass slides and stained with hematoxylin and eosin (H&E) for histological analysis.

### 2.13. In vivo synergistic therapy against orthotopic tumor-bearing mice

Cal27 cells were cultured to establish orthotopic oral squamous cell carcinoma (OSCC) model. 5 × 10^5^ Cal27 cells were suspended in 50 μL PBS, which were injected into the corpus linguae of BALB/c nude mice under anesthesia. Tumors grow steadily in tongue after one week, which marked the successful establishment of mice model of orthotopic tongue squamous cell carcinoma. Female BALB/c nude mice-bearing Cal27 tumors with a volume of about 20 mm^3^ were randomly divided into five groups (six mice in each group), including (1) control (treated with PBS), (2) PBS +808 nm laser irradiation (1.5 W cm^−2^), (3) SAF-SH (10 mg kg^−1^), (4) SAF-SNO (10 mg kg^−1^) and (5) SAF-SNO (10 mg kg^−1^) + 808 nm laser irradiation (1.5 W cm^−2^). The volume of the tumors and weight of the mice were measured every 2 days for 14 days period. The tumor volume was calculated according to the following formula: (tumor length) × (tumor width)^2^/2. The tumors weights were determined after synergistic treatment period. Subsequently, the tumor tissues were fixed in formaldehyde and embedded in paraffin. Paraffin-embedded tumor slices (~10 μm thickness) were cut and transferred onto a glass slide. After deparaffinization, the slides were boiled for 90 s in 0.01 mol sodium citrate buffer (pH 6.0) in pressure cooker for antigen retrieval. Then, the slides were allowed to cool for 5 min in the same buffer. After several rinses in PBS and pretreatment with blocking serum for 15 min, the slides were stained with H&E, TUNEL and Ki-67 for histological analysis. Furthermore, NO generation of tumor tissues was measured by NO-specific 4,5-diamino-N, N, N′, N′-tetraethylrhodamine (DAR-1) fluorescence probe. Briefly, the slides were stained with DAR-1 (20 µM) and the fluorescence intensity (excitation at 560 nm, emission at 595 nm) was measured using fluorescence spectrophotometer (F-4600, Hitachi, Ltd., Tokyo, Japan). Besides, employing the corresponding antibody to detect the apoptotic proteins (p53, Bax, Cytochrome c, cleaved Caspase-3) expression levels of tumor tissues by immunofluorescence staining was verify the mechanism of anti-tumor effect.

## 3. Results and discussion

### 3.1. Synthesis and characterization of SAF-SNO nanocatalysts

**Figure 1.**
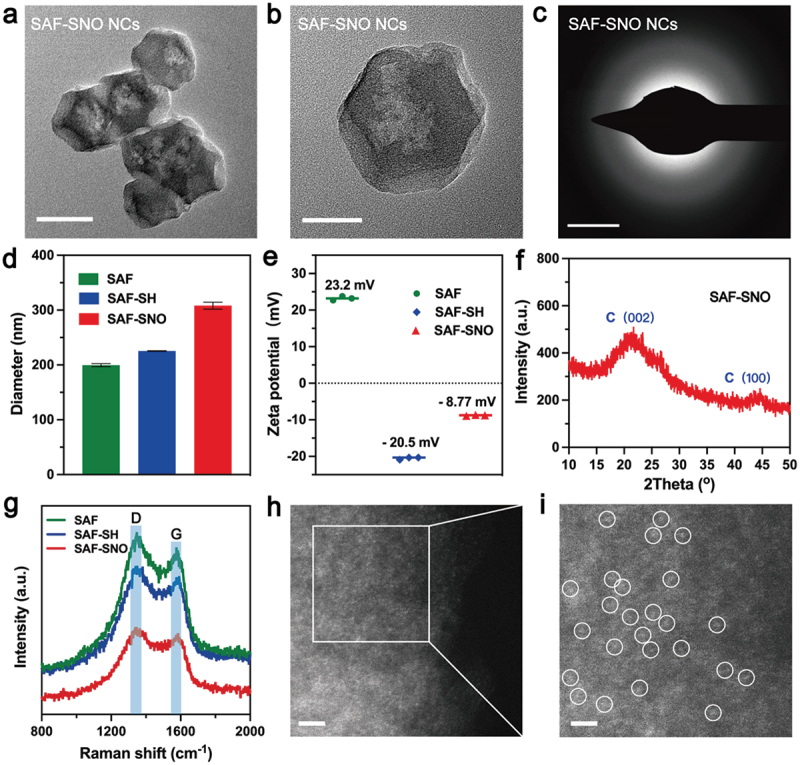
Synthesis and characterization of SAF-SNO NCs.

SAF-SNO nanocatalysts have been synthesized through the fabrication of SAF NCs with further modification with S-nitrosothiol (Scheme 1(a)). Initially, SAF NCs were synthesized by an ‘isolation-pyrolysis’ method [11]. Typically, zeolitic imidazolate frameworks (ZIF-8) with porous crystalline architecture formulate the encapsulation sites for the iron-containing precursor (Fe^III^ acetylacetone, Fe(acac)_3_) through one-step hydrothermal process. The obtained Fe(acac)_3_@ZIF-8 nanoparticles were then transferred to the quartz tubular furnace for pyrolysis under flowing argon, producing SAF NCs (Figure S1). For surface modification and functionalization of SAF NCs, 1,2-distearoyl-sn-glycero-3-phosphoethanolamine-N-[thiol (polyethylene glycol)] (DSPE-PEG-SH) was initially grafted onto the surface of SAF NCs via hydrophobic−hydrophobic interaction, yielding thiolated SAF-SH NCs with improved hydrophilicity and dispersibility. Then the thiolated SAF-SH NCs were further subjected to nitrosation through treatment of sodium nitrite (NaNO_2_) under weak acidic condition, featuring the covalent conjugation of S-Nitrosothiol (SNO) groups onto the nanocatalysts and yielding the nitric oxide producing SAF-SNO NCs. From the transmission electron microscopic (TEM) images, we found that SAF-SNO NCs exhibit typical dodecahedral geometry with an average size of approximately 120.8 nm (Figure 1(a,b)), showing relatively uniform morphology with an average size slightly larger than that of primitive SAF NCs (≈96.4 nm) (Figure S1). The presence of a diffuse halo in selected area electron diffraction pattern (SAED) indicates the amorphous architecture of the nanocatalysts (Figure 1(c)). Next, dynamic light scattering (DLS) method was employed to assay the average hydrodynamic diameter (Dh) and zeta potential of various synthetic nanocatalysts (SAF, SAF-SH, and SAF-SNO NCs), respectively (Figure 1(d,e)). Changes in zeta potential profile of the nanocatalysts provide evidences for successive conjugation of -SH group as well as the NO donor. Specifically, after DSPE-PEG-SH coating onto the surface of SAF NCs, the zeta potential of SAF NCs decrease from + 23.2 to −20.5 mV, suggesting the successful modification of -SH group. The chemical transformation of the thiol group then increases the surface potential to −8.77 mV. The changes of the sizes and zeta potential are derived from the valid surface engineering to significantly improve physiochemical stability. According to the X-ray powder diffraction (XRD) pattern, no crystalline peaks of iron but the broad bands located at 22° could be seen, assigning to the amorphous carbon, preliminarily indicating that the organic imidazolate framework has been well-carbonized into amorphous nitrogen-doped carbon substrate (Figure 1(f)). The Raman spectra of SAF-SNO NCs further reveal the amorphous architecture without crystalline structure (ID/IG = 1.08) (Figure 1(g)). In addition, no obvious differences of the waveforms between the Raman spectra of SAF, SAF-SH, and SAF-SNO NCs, implicating that the surface modification and functionalization did not affect the architecture of the amorphous substrate. Atomically dispersed single iron atoms were further recognized as the bright dots in high-angle annular dark-field (HAADF)-STEM images recorded by the Spherical aberration electron microscopy, indicating that the Fe(acac)_3_ precursors molecules have been pyrolyzed and isolated (Figure 1(h,i)).

(a, b) TEM images of SAF-SNO NCs. Scale Bar: (a) 100 nm and (b) 50 nm. (c) SAED pattern of SAF-SNO NCs. Scale bar: 5 nm^−1^. (d) Z-average hydrodynamic diameter of SAF NCs, SAF-SH and SAF-SNO NCs. (e) Zeta potentials of SAF NCs, SAF-SH and SAF-SNO NCs. (f) XRD pattern of SAF-SNO NCs. (g) FTIR spectra of SAF NCs, SAF-SH and SAF-SNO NCs. (h, i) HADDF-STEM images of SAF-SNO NCs. Scale bar: (h) 2 nm and (i) 1 nm.

**Figure 2.**
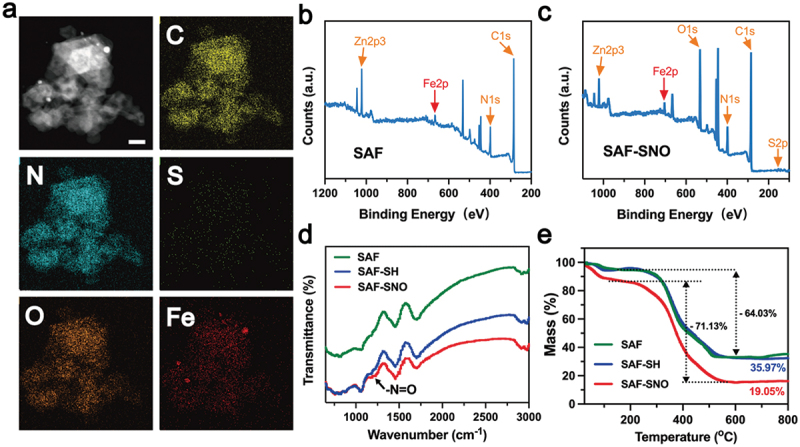
Characterizations of SAF-SNO NCs.

From the elemental mapping, homogeneous distributions of the C, N, O, S and Fe elements to the architecture of the nanocatalysts could be observed (Figure 2(a), Figure S2). We then employed XPS to investigate the elemental valences of SAF-SNO NCs. As compared to the primitive SAF NCs, the emerging elements of S and O in the spectra of SAF-SNO NCs validate the successful nitrosation onto the SAF NCs (Figure 2(b,c)). We next applied the Fourier transform infrared spectroscopy (FTIR) to identify the chemical vibrations of SAF-SNO. We observed the emergence of the characteristic peak of -N=O at 1190 cm^−1^, which is attributed to the functionalization of the -SNO group (Figure 2(d)). The amount of the -SNO functional groups was further evaluated by thermogravimetric analysis (TGA), from which the amount of SNO was determined to be as high as 14.92% (Figure 2(e)), presenting considerable potentials for NO gas therapy. The present results collectively indicate the successful engineering and functionalization of the SAF NCs towards NO-producing SAF-SNO NCs for favorable biomedical therapeutics.

(a) Elemental mapping (EDS-mapping) of SAF-SNO NCs. Scale bar: 50 nm. (b, c) XPS spectra of (b) SAF NCs and (c) SAF-SNO NCs. (d) Raman spectra of SAF NCs, SAF-SH and SAF-SNO NCs. (e) Thermogravimetric analysis (TGA) of SAF NCs, SAF-SH and SAF-SNO NCs.

### 3.2. Fenton catalytic performance of SAF-SNO NCs

**Figure 3.**
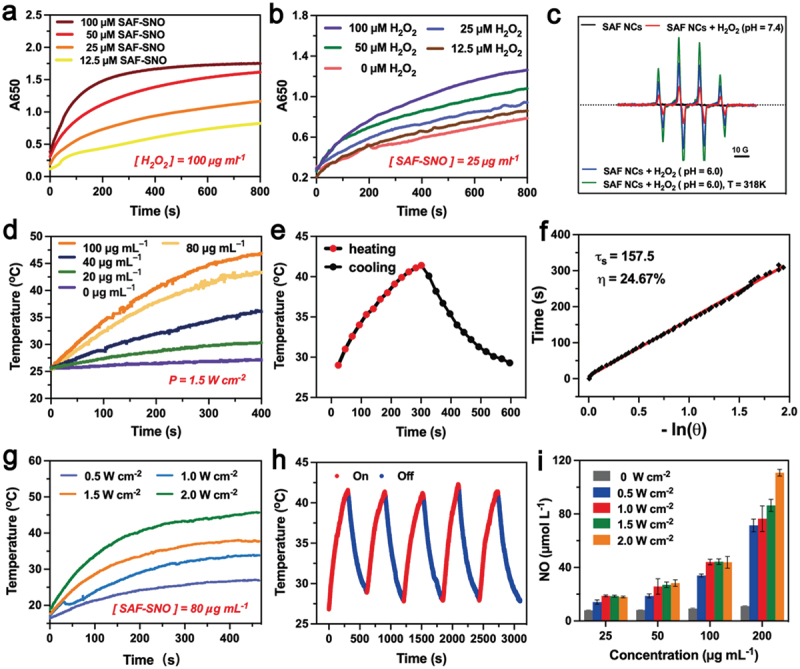
Catalytic, photonic hyperthermia and hyperthermia-triggered NO release performances of SAF – SNO NCs.

Ferrous ions have been demonstrated to be capable in initiating potent homogeneous Fenton reaction to produce hydroxyl radicals (•OH) in aqueous acidic media with strong oxidative stress. For non-iron releasing SAF NCs, corresponding heterogeneous Fenton reaction could be initiated onto the single iron sites of the nanocatalysts. First, we evaluated the heterogeneous Fenton catalytic performance of SAF-SNO NCs by the chromogenic kinetic test using 3,3′,5,5′-tetramethylbenzidine (TMB). In the presence of TMB, H_2_O_2_ and the acidic buffer, addition of the SAF-SNO NCs with varied concentrations immediately turned the assay solution into blue appearance, recording a prominent time-dependent optical absorption at 650 nm, indicating that SAF-SNO NCs possess the Fenton catalytic effect in decomposing H_2_O_2_ into oxidative hydroxyl radicals (Figure 3(a,b), Figure S3). In addition, characteristic radical signals of •OH were detected in ESR spectra when 5,5-dimethyl-1-pyrroline N-oxide (DMPO) was served as the radical trapping agent against •OH, upon the addition of SAF-SNO NCs and H_2_O_2_ (100 μM) in acidic buffer condition. Weakened spectral signals could be observed when the reaction assay was suspended in neutral buffer, while enhanced spectral signals could be recorded under elevated temperature from 26°C to 45°C, demonstrating the hyperthermia-enhanced Fenton kinetics with bulky yield of the •OH radicals (Figure 3(c)).

(a) The time-course absorbance of TMB upon the additions of varied concentrations of SAF-SNO NCs and H_2_O_2_ of 100 μg mL^−1^. (b) The time-course absorbance of TMB for solutions containing SAF-SNO NCs (25 μg mL^−1^) and H_2_O_2_ of varied concentrations. (c) ESR spectra of SAF-SNO NCs recorded under indicated conditions using DMPO as the radical trapper. (d) Photothermal-heating curves of SAF – SNO NCs solutions of varied doses under 808 nm laser irradiation (*p* = 1.5 W cm^−2^). (e) Photonic heating and cooling profile of SAF – SNO NCs solution (80 μg mL^−1^) under 808 nm laser irradiation at 1.0 W cm^−2^ and laser-off. (f) Time constant for heat transfer using the linear time data from the cooling stage. (g) Photothermal-heating curves of SAF – SNO NCs solution (80 μg mL^−1^) on elevated power densities of 808 nm laser irradiation. (h) Recycling heating-cooling curves of SAF – SNO solution (80 μg mL^−1^) under 808 nm laser irradiation (1.0 W cm^−2^) for five laser on/off cycles. (i) Quantitative measurements of NO releases from SAF-SNO under varied concentrations and different power densities.

### 3.3. Hyperthermia-responsive NO generation by SAF-SNO NCs

The functioning -SNO groups feature with thermal-responsiveness in decomposition for NO generation. In the present design, NIR laser (808 nm) was employed to trigger the hyperthermia performance of SAF-SNO NCs, decomposing the functioning -SNO groups for NO generation. At first, the photonic hyperthermia conversion performance was investigated by the application of the 808 nm NIR laser at varied concentrations and NIR power densities. The elevated temperature profile as well as the hyperthermia images of SAF-SNO aqueous solution were recorded by the IR thermal imaging camera (Figure S4). Specifically, the temperature of the SAF-SNO solution at the concentration of 80 µg mL^−1^ was increased to as high as 46.9°C under the irradiation of 808 nm NIR (1.5 W cm^−2^) for 5 min, providing the basis for potential thermal decomposition of the -SNO groups for NO liberation (Figure 3(d)). The photothermal-conversion efficiency (η) was calculated to be 24.67% (Figure 3(e,f)), indicating that NIR irradiation could efficiently and rapidly convert photon energy into heat in the presence of SAF-SNO NCs. By adjusting the laser power densities, the elevated temperature values of the solution were determined to be 10.5, 16.6, 20.3 and 26.9°C, corresponded to the power densities of 0.5, 1.0, 1.5 and 2.0 W cm^−2^, respectively (Figure 3(g)). We then evaluated the photonic hyperthermia stability of SAF-SNO NCs. By repeating the heating and cooling cycles with 808 nm NIR irradiation (5 min heating, 5 min cooling), no significant attenuation in both heating rate and maximal amplitude could be observed (Figure 3(h)). These results indicate the structural and photonic hyperthermia stabilities of SAF-SNO NCs under NIR irradiation, benefiting the overall therapeutic process based on the catalytic therapy, hyperthermia and heat-enabled gas therapy.

Next, the liberation of NO molecules from SAF-SNO NCs was quantitatively measured using the commercial Griess reagent, a detection probe that measures the concentration of solution nitrite ions (NO^2−^) as the nonvolatile products when NO was transformed within aqueous medium, yielding characteristic optical absorption at 540 nm. For SAF NCs and SAF-SH NCs under laser irradiation (808 nm, 5 min), no apparent color change of the solution could be observed, implicating the inability for these NCs to generate NO. While for SAF-SNO NCs, the appearance of SAF-SNO solution gradually turned from black to red after the addition of the Griess reagent and subsequent laser irradiation, demonstrating that NIR irradiation could effectively trigger the decomposition of the covalently conjugated -SNO groups and ultimately NO liberation from SAF-SNO NCs. Specifically, 45.53 µM of NO was detected when 100 µg mL^−1^ of SAF-SNO NCs were irradiated with 808 nm laser. Without laser irradiation, only 7.94 µM of NO could be produced. The amount of NO liberation substantially increased as the elevated concentration of SAF-SNO NCs (Figure 3(i)). We also found that the NO liberation features with a dependent manner to the power density parameters of the NIR laser. The above results demonstrate that the SAF-SNO NCs feature with prominent hyperthermia effect upon the irradiation of NIR laser, capable of decomposing the -SNO groups for NO liberation effectively.

### 3.4. In vitro synergistic therapy against Cal27 cancer cells

Based on the excellent nanocatalytic performance and hyperthermia-enabled NO liberation properties, we then proceed on the investigation of the *in vitro* synergistic therapy against human squamous carcinoma cell line Cal27. The cytotoxicity profile of the synthetic NCs were first evaluated by a standard Cell Counting Kit-8 (CCK-8) assay after the co-incubation of SAF-SH and SAF-SNO NCs with Cal27 cells for 24 h. Cells treated with both SAF-SH NCs and SAF-SNO NCs show highly biocompatible viability toward Cal27 cells even at the concentration of the NCs reached as high as 200 µg mL^−1^ (RCV = 82.8%) (Figure S5(a)). Besides, treatment of tumor cells with either acidic culture medium (pH = 6.0) or H_2_O_2_ (100 μM) showed negligible influence on the proliferation of Cal27 cancer cells (Figure S5(b)). However, the survival of Cal27 cells treated with SAF-SNO NCs was generally a slightly lower than the cells treated with SAF-SH NCs, probably due to the minor production of NO within tumor cells.

Then, the cellular uptake of SAF-SNO NCs by Cal27 cells was evaluated by laser scanning confocal microscopy (LSCM). The green fluorescence signal from FITC-labeled SAF-SNO NCs and the blue fluorescence signal from 4’,6-diamidino-2-phenylindole (DAPI)-labeled cell nucleus demonstrated the effective SAF-SNO NCs location, certifying that SAF-SNO NCs were engulfed by Cal27 cells via endocytosis and the quantity was accumulated with the incubation time (Figure S6).

**Figure 4.**
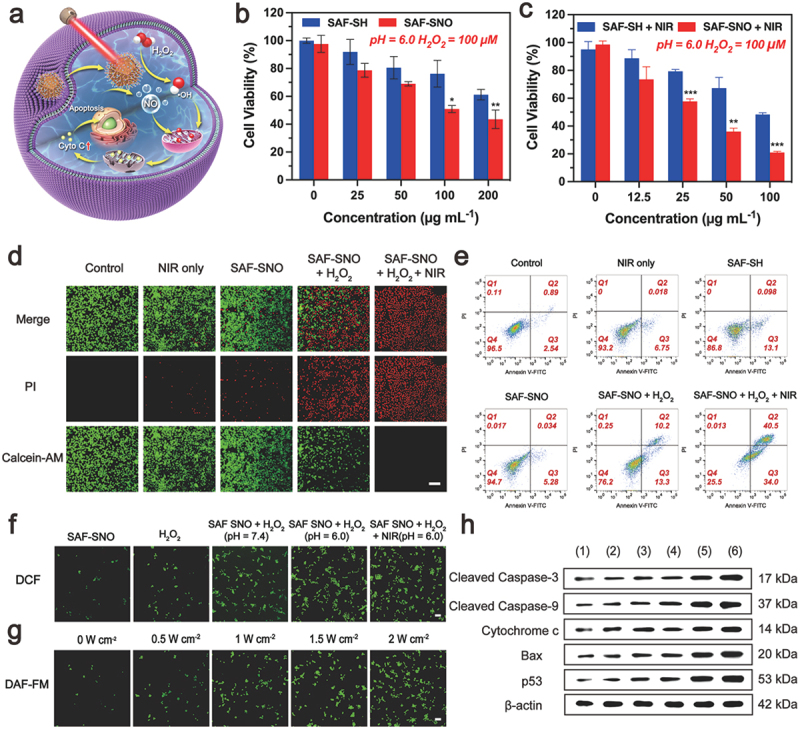
In vitro synergistic therapy against Cal27 cells.

When SAF-SNO NCs were endocytosized by Cal27 tumor cells, these NCs could produce large quantities of toxic •OH when exposed to intracellular H_2_O_2_, initiating the heterogeneous Fenton catalytic tumor therapy within the tumor sites. In addition, under the stimulation of the NIR irradiation, photonic hyperthermia could be generated by SAF-SNO NCs in the meantime, locally decomposing the S-nitrosothiol group for NO liberation, achieving the synergistic therapeutics of the nanocatalytic therapy and gas therapy (Figure 4(a)). The sequential comprehensive synergistic therapeutic efficacy of SAF-SNO NCs against malignant tumor cells (Cal27) was investigated. When Cal27 cells were treated with varied doses of SAF-SH and SAF-SNO NCs along with the addition of 100 μM H_2_O_2_ under an acidic medium (pH = 6.0, mimicking the physiochemical feature of TME), apparent proliferation inhibition effect (RCV = 61.14%, 36.86%, respectively) was observed when the concentration of NCs were up to 200 µg mL^−1^ (Figure 4(b)). Such cytotoxicity result was attributed to the performance of the nanocatalytic therapy triggered by the TME-responsive heterogeneous Fenton catalytic reaction by SAF NCs. The cell survival rates gradually decreased as the increasing SAF-SNO NCs concentrations as well as the NIR laser power densities (Figure 4(c), Figure S7). Notably, SAF-SNO NCs presented a stronger effect in killing cancer cells as compared to that of SAF-SH NCs, especially at the concentration of 100 µg mL^−1^. The cell viability was remarkably reduced to 20.96% for cells in SAF-SNO NCs + H_2_O_2_ + NIR group, while nearly 48.29% of cells were still survived upon SAF-SH NCs + H_2_O_2_ + NIR treatment, suggesting the thermoresponsive NO liberation performance and their anti-tumor induction. Based on the cytotoxicity study, a nanocatalysts dosage of 100 µg mL^−1^ was selected as the working concentration in the cellular experiments that follow.

(a) Schematic illustration of synergistic therapy by SAF – SNO at the cell level. (b, c) Cal27 tumor cell viability assays of SAF-SH and SAF-SNO NCs under different conditions including: (b) adding H_2_O_2_ into acidified (pH = 6.0) culture media and (c) with extra laser irradiation exposure (1.5 W cm^−2^). Statistical significances were calculated via Student’s t test, *p < 0.05, **p < 0.01, ***p < 0.001 and n.s. for non-significant. (d) Confocal microscopic images for cells stained with Calcein-AM (green) and PI (red) after different treatments (PBS, NIR only, SAF-SNO, SAF – SNO + H_2_O_2_, SAF – SNO + H_2_O_2_ + NIR). Scale bars: 100 μm. (e) Flow cytometry quantification assay on apoptosis rate of Cal27 cells after different treatments (PBS, NIR only, SAF-SH, SAF-SNO, SAF – SNO + H_2_O_2_, SAF – SNO + H_2_O_2_ + NIR). (f) Confocal microscopic images for cells treated with SAF-SNO, H_2_O_2_, SAF-SNO + H_2_O_2_ (PH = 7.4), SAF-SNO + H_2_O_2_ (PH = 6.0), SAF-SNO + H_2_O_2_ (PH = 6.0) + NIR using DCFH-DA cell-staining. Scale bar: 100 μm. (g) Intracellular detection of NO release in Cal27 cells after coincubation with SAF – SNO followed by laser irradiation at different power densities (0, 0.5, 1.0, 1.5, and 2.0 W cm^−2^). Scale bar: 100 μm. (h) Western blot analyses of the p53, Bax, Cytochrome c, cleaved-caspase-3, and cleaved-caspase-9 protein expressions in cancer cells subject to different treatments: (1) Control, (2) NIR only, (3) SAF-SH, (4) SAF-SNO, (5) SAF – SNO + H_2_O_2_, (6) SAF – SNO + H_2_O_2_ + NIR.

To further determine the *in vitro* lethality of SAF-SNO NCs on tumor cells by synergistic therapeutic modality, viable and dead Cal27 cancer cells were visualized by fluorescence assay through confocal laser scanning microscopic (CLSM) imaging of pre-treated cells that were co-stained with Calcein-AM (green fluorescence, viable cells) and PI (red fluorescence, dead cells). As compared to the cells in the untreated control group, Cal27 cells irradiated with 808 nm laser alone showed strong green fluorescence signals, indicating non-toxicity during laser irradiation. When SAF-SH NCs or SAF-SNO NCs were supplemented with H_2_O_2_, enhanced cytotoxicity could be observed, which is attributed to the heterogeneous Fenton catalytic performance enabled by SAF NCs. In addition, a major red fluorescence could be observed on the microscopic image of cells treated with SAF-SNO + H_2_O_2_ + NIR, suggesting that the prominent cell killing effect is achieved by the synergistic nanocatalytic therapy and gaseous NO therapy (Figure 4(d)). We also employed flow cytometric assays to explore the apoptosis and necrosis of Cal27 cells after different treatments. It could be found that the ratios of apoptotic cells in SAF-SNO NCs + H_2_O_2_ and SAF-SNO NCs + H_2_O_2_ + NIR groups were significantly higher than that of the other four groups. The apoptotic cell populations reached 23.5% and 74.5%, respectively (Figure 4(e), Figure S8), which signifies the prominent performance of the synergistic therapy upon NIR laser irradiation. These results confirm that the synergistic therapy can efficiently trigger the apoptosis of cancer cell, combating malignant tumor effectively.

We next gain deeper insights into the kill effects in cellular manner. Intracellular oxidative stresses are conceived as the primary factor in generating the cytotoxicity. Intracellular generation of •OH via TME-responsive Fenton reaction were detected by the fluorescence signal intensities of tumor cells stained by •OH-sensitive dye (2′,7′-dichlorofluorescein diacetate, DCFH-DA). From the confocal microscopic images, we found that deem green fluorescence in the cells of untreated group with low generation of •OH inside the tumor cells. The fluorescence intensity apparently increased when the cells were treated with SAF-SNO NCs + H_2_O_2_ (100 μM) in acidic buffer. Significantly, bright green fluorescence could be observed for cells treated with NIR laser irradiation, implying the mild hyperthermia could effectively enhance the process of TME-responsive Fenton reaction against the tumor cells (Figure 4(f), Figure S9). Next, we evaluated the intracellular NO generation from SAF-SNO NCs using the NO-specific dye 3-amino-4-aminomethyl-2′,7′-difluorescein, diacetate (DAF-FM DA). We found that a large number of NO was released into the Cal27 cancer cells when treated with NIR laser irradiation (Figure 4(g), Figure S10(a)). By adjusting the laser power densities, the increased amount of NO released were approximately 6, 10, 15 and 22 folds higher than those in the absence of the laser, corresponded to the power densities of 0.5, 1.0, 1.5 and 2.0 W cm^−2^, respectively (Figure S10(b)). The amount of NO increased along with the elevated NIR power density, indicating that SAF-SNO NCs could release NO *in situ* under NIR laser irradiation and the quantity of NO could be well controlled by adjusting the NIR power density.

We then sought to investigate the underlying molecular mechanism of cell death induced by SAF-SNO NCs. Apoptosis has been generally recognized as a programmed cell death characterized by distinct morphological changes and activation of specific caspase proteins through specific pathways associated with mitochondria [33]. The mitochondrial membrane potential is an important parameter of mitochondrial function and has been used as an indicator of cell health. 5,5′,6,6′-tetrachloro1,1′,3,3′-tetramethylbenzimidazolylcarbocyanine iodide dye (JC-1) has been used to monitor mitochondrial integrity. JC-1 forms aggregates (red fluorescence) when it enters intact mitochondria, while existing as a monomer (green fluorescence) under the condition of mitochondrial membrane potential collapse. The increased characteristic monomer of JC-1 showed SAF-SNO NCs initiated the loss of mitochondrial membrane potential and disrupted mitochondrial integrity (Figure S11). Furthermore, cytochrome c was detected as the marker of mitochondria damage and apoptosis activation [34]. We observed major green fluorescence signal of cytochrome c immunofluorescence, identifying that cytochrome c is uniformly dispersed in the cells treated with SAF-SNO NCs + H_2_O_2_ under NIR irradiation (Figure S12), indicating the complete release of cytochrome c from mitochondria into cytosol under the stimulation of apoptotic signals. The released cytochrome c could further activate the caspase-3-mediated apoptosis pathway. To understand the mechanism of tumor growth inhibition, the expression levels of apoptosis-associated proteins (p53, Bax, Cytochrome c, cleaved caspase-9, cleaved caspase-3) were evaluated by western blotting. The results exhibited that the expression of these proteins in groups (5) and (6) were upregulated remarkably, but the protein expression levels of the (6) group were higher than the (5) group, while low expression profiles could be observed for other treatment groups (Figure 4(h), Figure S13). P53 is a tumor suppressor protein that induces apoptosis, cell cycle arrest, or senescence in response to distinct stimulation [35,36]. Bax, a part of the Bcl-2 family proteins and the major pro-apoptosis target genes of p53, is critical regulators of apoptosis and its primary site of action is on the outer mitochondrial membrane (OMM) [37]. These two proteins are linked by a mitochondrial apoptotic pathway [38]. It has been documented that if cells are suffering severe oxidative stresses, they would shift to a pro-death mode and will facilitate the induction of apoptosis [39]. At the molecular level, under a variety of cell-death-inducing conditions, abundant ROS and NO are induced by SAF-SNO NCs + H_2_O_2_ + NIR, p53 rapidly moves to the mitochondria and trigger the pro-apoptotic Bax proteins on the OMM, leading to the permeabilization of OMM and subsequent release of apoptotic marker cytochrome c into the cytosol that ultimately trigger a cascade of reaction with the assistance of caspases [40]. The initiator caspase of the intrinsic pathway, caspase-9, enabling autoactivation, which in turn activates the series of downstream of cascade to activate the caspase-3, an effector caspase involved in cell death [41,42]. Collectively, by evaluating the expressions of the key proteins through western blotting, we validated that the therapeutic mechanism of SAF-SNO NCs is associated with a p53-initiated mitochondrial-oriented apoptotic pathway for tumor destruction.

### 3.5. In vivo synergistic therapeutics of SAF-SNO NCs

Encouraged by the outstanding performance of synergistic efficacy of cytotoxic impacts against malignant tumor cells *in vitro*, *in vivo* tumor inhibition effect was further investigated with the orthotopic Cal27 tumor-bearing BALB/c mice. All animal experiments have been approved by the institutional animal care and committee of Shanghai Ninth People’s Hospital, Shanghai Jiao Tong University School of Medicine (SH9H-2019-A176-1). Initially, *in vivo* biosafety of nanocatalysts was an important prerequisite to investigate for guaranteeing the safety for further experiments. Healthy female BALB/c nude mice were randomly divided into three groups, including control group, SAF-SH NCs (20 mg kg^−1^, single dose, i.v.) group and SAF-SNO NCs (20 mg kg^−1^, single dose, i.v.) group. Evaluations including the time-course body weight change, the blood biochemistry tests, and the hematoxylin and eosin (H&E) histologic examination of major organs were comprehensively conducted for biocompatibility assessments. It turns out that negligible weight variations were recorded between the control group and the treatment groups (Figure S14(a)). The statistical data from hematological examinations and hepatic/renal function evaluations show no abnormalities in all assayed physiological function markers (Figure S14(b)). No apparent organ damages could be indicated in the H&E staining of major organs of mice in different groups (Figures S15), demonstrating that negligible systemic side effect of the treatments of mice. Cy5.5-labeled SAF-SNO NCs were applied to track the nanocatalysts *in vivo*. In 24 h post-injection, mice were euthanatized and fluorescence imaging techniques were applied to monitor the biodistribution of SAF-SNO NCs at specific points in time (Figure S16).

In addition, inductively coupled plasma mass spectrometry (ICP-MS) was employed for quantitative analysis of SAF-SNO biodistribution *in vivo* (Figure S17). These results show that SAF-SNO NCs can be effectively accumulated in tumor xenografts in 8 h after intratumoral injection, which guarantees the sufficient therapeutic window for NIR irradiation against malignant tumors. In 48 h post-injection, SAF-SNO NCs are mainly excreted through the liver and kidney, demonstrating the satisfactory *in vivo* elimination kinetics. These results indicate that the excellent biocompatibility of the fabricated SAF-SNO NCs, presenting high potentials for further *in vivo* therapeutics and clinical transformation.

**Figure 5.**
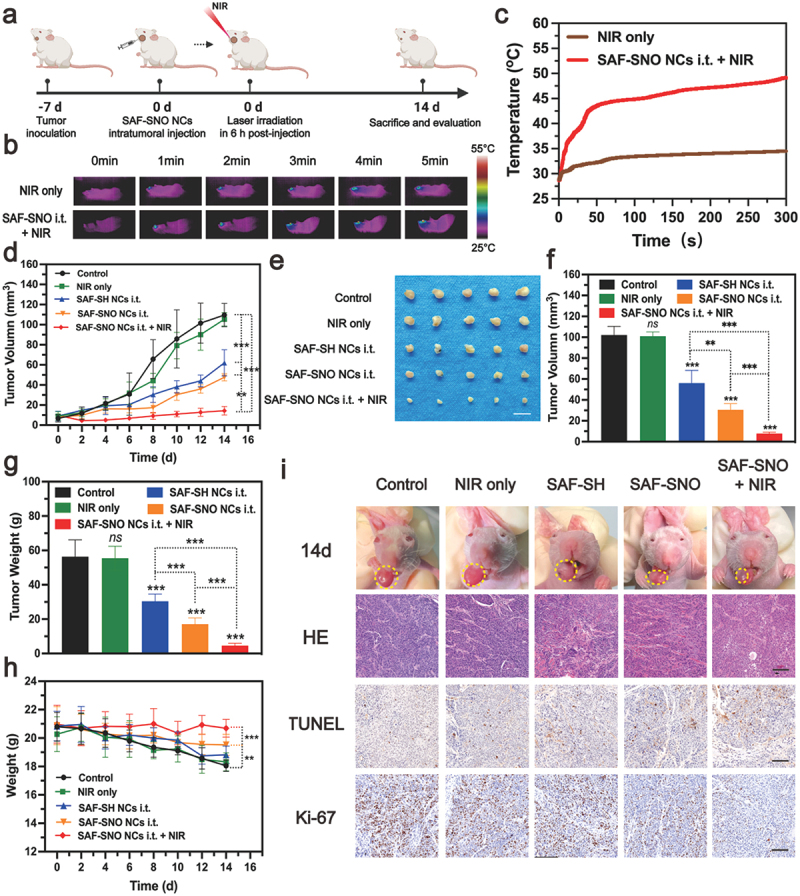
In vivo tumor growth inhibitions by SAF-SNO NCs on orthotopic tumor-bearing Mice.

Following the extensive evaluation of the *in vivo* biosafety of the nanocatalysts, the synergistic therapeutic performance of SAF-SNO NCs was then evaluated *in vivo* within 14 days of timeframe (Figure 5(a)). Initially, to establish the orthotopic murine oral carcinoma model, Cal27 cells (5 × 10^5^ cells) were directly injected into the corpus linguae of the BALB/c nude mice under anesthesia. The xenografts were allowed to grow for 7 days to reach an average of tumor volume of 20 cm^3^. These mice were randomly divided into five groups (*n* = 5 in each group): (1) control, (2) NIR irradiation only, 3) SAF-SH NCs (i.t.), 4) SAF-SNO NCs (i.t.), 5) SAF-SNO NCs (i.t.) + NIR. The injection doses for SAF-SH and SAF-SNO NCs were equally 10 mg kg^−1^ while the power density of the NIR irradiation treatment was set as 1.5 W cm^−2^ for 5 min. Real-time monitoring of the temperature profile was recorded using an infrared thermal imager during the laser irradiation process (Figure 5(b)). According to the heating curve, apparent localized hyperthermia generation in the tumor region from 28.6°C to 50.1°C could be observed for the xenograft of mice in the SAF-SNO NCs i.t. + NIR group, while the tumor-site temperature of the NIR group increased slightly from 28.8°C to 34.6°C (Figure 5(c)). These findings showed the SAF-SNO NCs possesses distinct NIR absorption with favorable photonic hyperthermia conversion efficiency in tumor site, decomposing the -SNO groups for NO liberation intratumorally.

(a) Treatment schedule for SAF-SNO NCs-based synergistic therapeutic modality. (b, c) *In vivo* (b) infrared imaging photos for mice under exposure to 808 nm NIR irradiation for 5 min, 4 h after the SAF-SNO NCs injection (saline instead for NIR only group) intratumorally, and (c) localized tumor temperature curves. (d) *In vivo* time-dependent tumor-growth curves of mice under different treatments (*n* = 5, mean ± SD): control group, NIR only, SAF-SH NCs i.t. (10 mg kg^−1^), SAF-SNO NCs i.t. (10 mg kg^−1^) and SAF-SNO NCs i.t. (10 mg kg^−1^) + 808 nm laser irradiation for 5 min. (e-g) Digital photographs (e), terminal tumor volume (f) and weight (g) of dissected tumors from differently treated groups in 14 days of treatments. Scale bar: 1 cm. (h) Time-dependent body-weight curves of nude mice (*n* = 5, mean ± SD) after various treatments (i) Digital images and histological assessments (including H&E staining, TUNEL assay, and antigen Ki-67 immunofluorescence staining) of tumor regions of orthotopic tumor-bearing mice at the 14th day after different treatments. Scale bar: 100 μm. Statistical significances were calculated via Student’s t test, *p < 0.05, **p < 0.01, ***p < 0.001 and n.s. for non-significant.

The body weights of mice and xenograft dimension profile (length × width^2^/2) in control and different treatment groups were weighted and collected every 2 days in the evaluation timeframe. From the tumor development profiles, no significant difference in tumor growth could be observed when compared the control group with the NIR group, demonstrating that the hyperthermia alone negligibly affects the tumor growth. We also found that significant growth of tumor volumes in the groups of control, NIR only, SAF-SH NCs i.t., and SAF-SNO NCs i.t., while the groups of SAF-SNO NCs i.t. + NIR showed the best tumor growth inhibitory efficacy among all the treatment groups (Figure 5(d)). At the end of the evaluation, all mice were euthanized with their xenograft and major organs dissected. From the digital photographs of the dissected tumors, although mice in the group of SAF-SH NCs and SAF-SNO NCs only exhibited a certain inhibitory effect on tumor growth due to the *in vivo* heterogeneous Fenton catalysis of endogenous H_2_O_2_ to toxic •OH for tumor killing, the excellent therapeutic outcome was recorded based on the synergistic therapeutics by Fenton catalytic therapy and NO gaseous therapy (Figure 5(e–g)). For mice in control and all the treatment groups, body weight loss was frequently observed, possibly due to their difficulty in accessing water and food during the progression of tongue tumors. However, the body weight of mice in the SAF-SNO NCs i.t. + NIR group after the therapeutics substantially recovered as compared to the other groups, further indicating that the amelioration of the oral function due to the relief of tumor burden after the orthotopic therapeutics (Figure 5(h)).

For pathological inspections, the dissected tumor xenografts from different treatments of mice were further cut into ultrathin sections for immunohistochemical evaluations, including H&E, antigen Ki-67 (a biomarker of cell proliferation), and terminal deoxynucleotidyl transferase-mediated dUTP-biotin nick end labeling (TUNEL, a marker of apoptotic cells) staining (Figure 5(i)). The images of H&E-stained tumor sections showed that the SAF-SNO NCs i.t. + NIR induced prominent cellular damage against the tumor cells. A substantially lower Ki-67 fluorescence signal and a higher TUNEL fluorescence signal were detected in the microscopic images from SAF-SNO NCs i.t. + NIR treatment group than that in the other groups, manifesting remarkable tumor suppression after effective therapeutics enabled by SAF-SNO NCs. To investigate NO-induced release *in vivo*, NO-specific fluorescence probe DAR-1 was conducted in mice bearing tumors after different treatments. A significant enhanced fluorescent signal of DAR-1 in SAF-SNO NCs i.t. + NIR-treated tumors was observed, suggesting that NO could be efficiently released from SAF-SNO NCs in tumors (Figure S18). Moreover, immunofluorescent technique was employed to detect the expression profile of apoptotic proteins (p53, Cytochrome c, cleaved caspase 3) inside tumor. From the microscopic images, strong red fluorescence could be observed in SAF-SNO NCs i.t. + NIR group, while the tumor tissue of other groups exhibited weak fluorescence (Figure S19). These observations demonstrate that the anti-tumor synergistic therapy effectively trigger cell apoptosis for tumor destruction. Collectively, the above results confirm that the combination of nanocatalytic therapy and NO gaseous therapy could achieve prominent therapeutic efficacy against tumor with highly biocompatibility both *in vitro* and *in vivo*.

## 4. Conclusion

In summary, we present an appealing synergistic therapeutic modality combining nanocatalytic ROS and NO gas productions against local OSCC based on the single-atom Fe nanocatalysts engineered with NO donor. The nanocatalyst is able to inhibit orthotopic OSCC tumor efficiently by multiple and synergistic effects under mild hyperthermia, especially the substantial catalytic hydroxyl radical production in response to specific tumor microenvironment as well as the photoresponsive ‘on-demand’ transformation for NO liberation. Both *in vitro* and *in vivo* outcomes have presented that SAF-SNO NCs exhibit outstanding therapeutic efficacy toward cancer cells via inducing the mitochondrial-oriented apoptotic pathway. An overall tumor suppression rate of 85.57% has been achieved *in vivo* via one dose i.t. SAF-SNO NCs injection under laser irradiation. Furthermore, the SAF-SNO NCs also feature excellent biocompatibility and biosafety, providing a foundation for further potential clinical transformation. Collectively, this work not only provides a promising cancer therapeutic modality for orthotopic OSCC, but also shows promising potentials for clinical applications against oral cancers.

## Supplementary Material

Supplemental Material
