# Supplementary material for: Nanocatalytic NO gas therapy against orthotopic oral squamous cell carcinoma by single iron atomic nanocatalysts
Source: Sci Technol Adv Mater. 2024 Jun 28;25(1):2368452. doi: 10.1080/14686996.2024.2368452 (PMC11238653; doi:10.1080/14686996.2024.2368452)
Supplement: Supplemental Material [file TSTA_A_2368452_SM1959.docx]

Supporting Information of

**Nanocatalytic NO gas therapy against orthotopic oral squamous cell carcinoma by single iron atomic nanocatalysts**

Yuting Xie^a^, Jiaxin Zuo^a^, Angang Ding^a^, Ping Xiong^a^*

^a^ Department of Ultrasound, Shanghai Ninth People’s Hospital, Shanghai Jiaotong University School of Medicine, Shanghai 200011, P. R. China

E-mail: xiongp@shsmu.edu.cn

**Supplementary Figures**


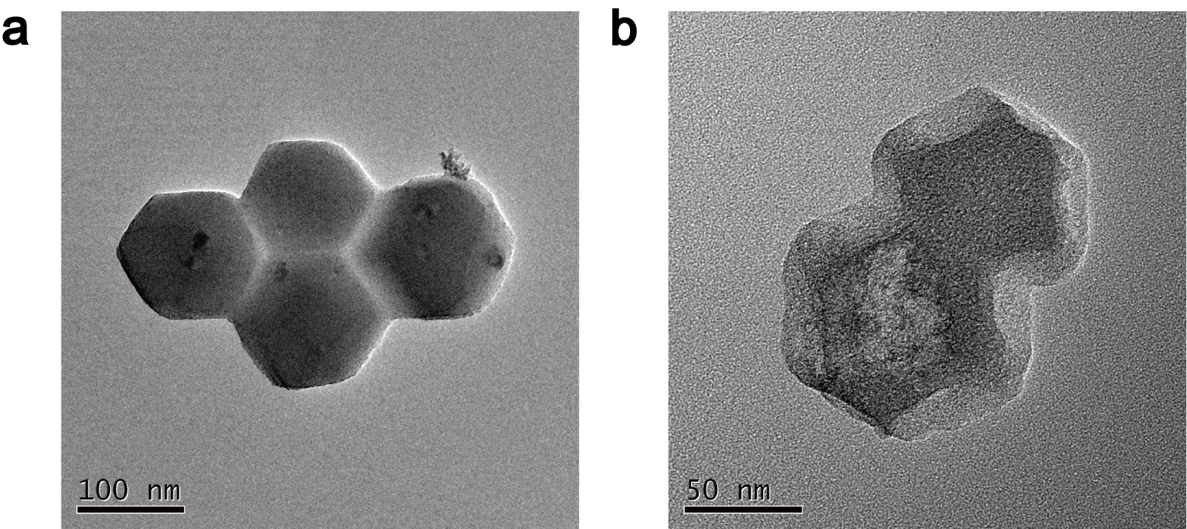


**Figure S1**.TEM image of (a) Fe(acac)_3_@ZIF-8 NPs and (b) SAF NCs.


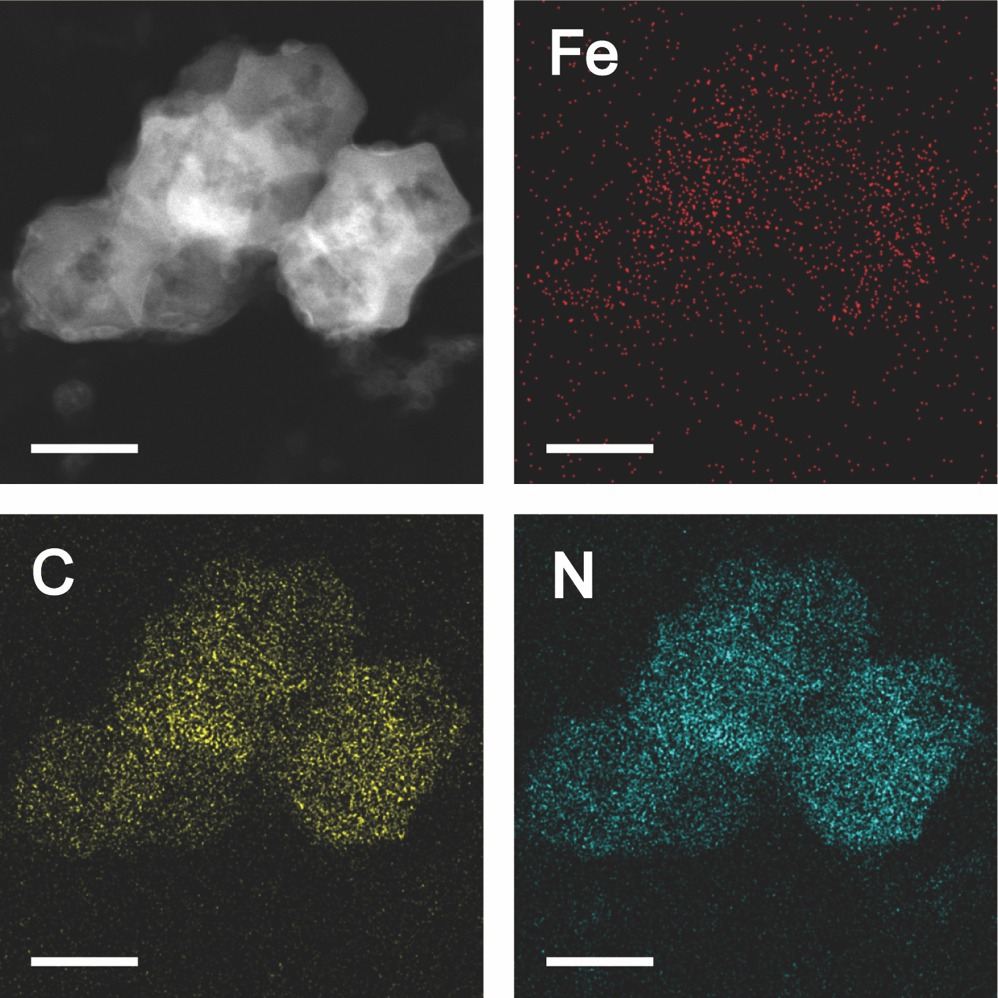


**Figure S2.** Corresponding elemental mapping (EDS-mapping) of SAF NCs. Scale bar: 50 nm.


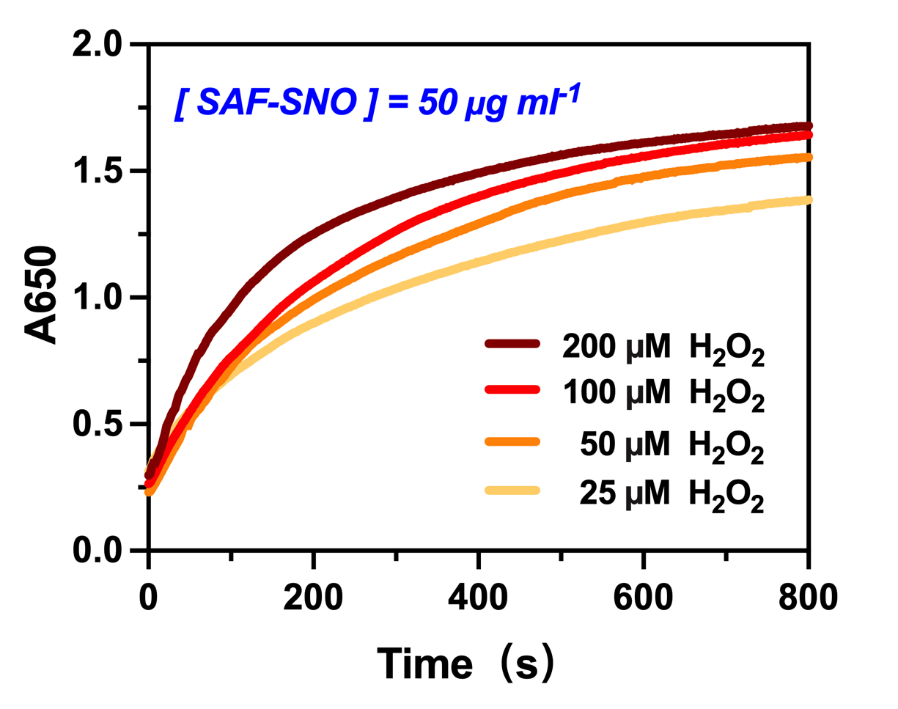


**Figure S3.** The absorbance of TMB upon the addition of SAF-SNO NCs (50 μg mL^−1^) and varied concentrations of H_2_O_2_.


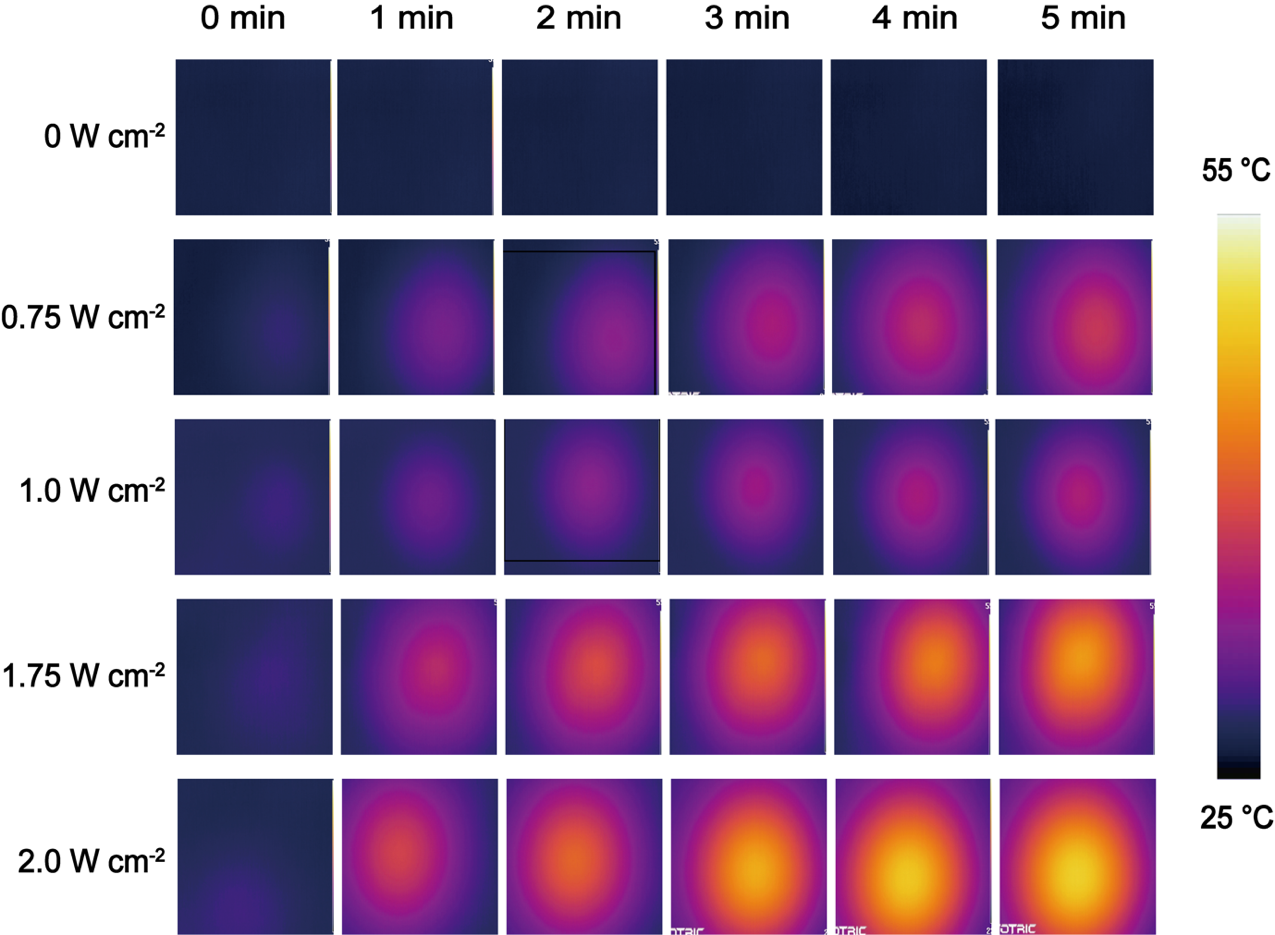


**Figure S4.** The photothermal images captured by the IR thermal imaging camera of SAF-SNO solution under 808 nm laser irradiation with varied laser power densities at different time points.


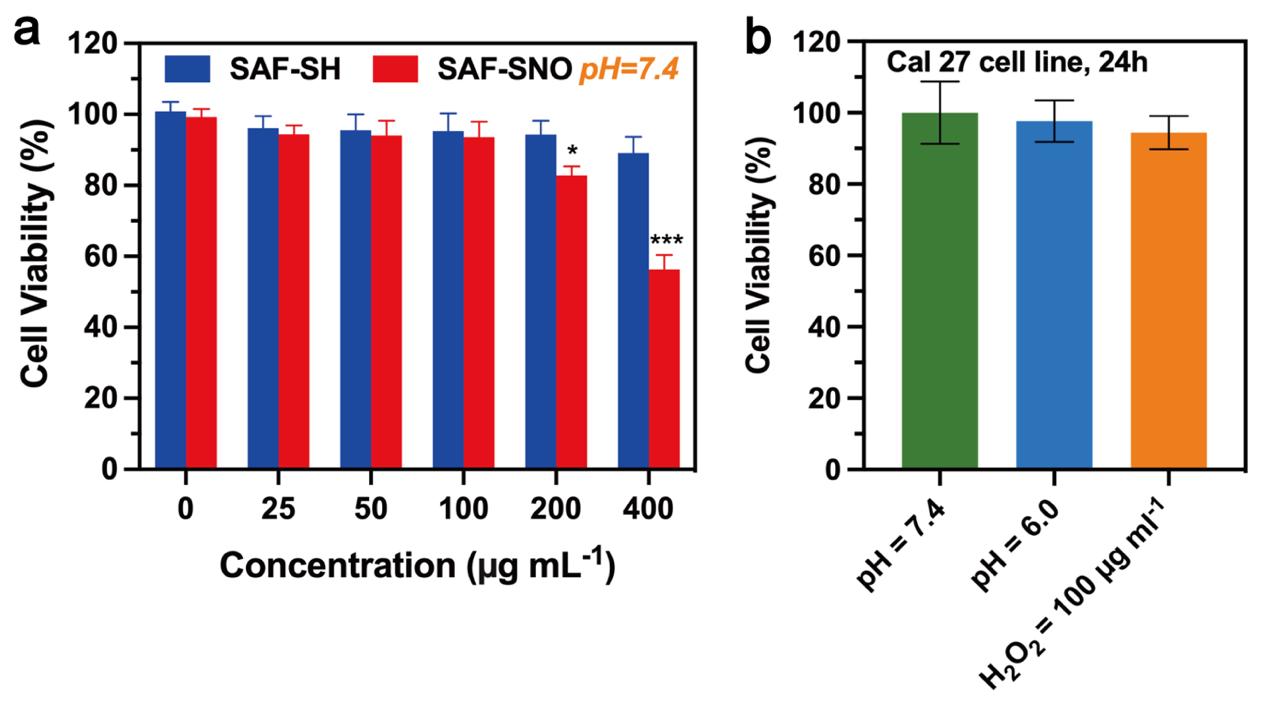


**Figure S5.** In vitro cytotoxicity assessment of Cal27 cells. (a) Cell viability of Cal27 treated with varied concentrations of SAF-SH and SAF-SNO NCs under normal neutral condition (pH = 7.4). (b) Cell viability of Cal27 under different treatments including: normal neutral condition (pH = 7.4), acidic environment (pH = 6.0) and H_2_O_2_ (100 μg ml^-1^) supplementation. (*p<0.05, **p<0.01, ***p<0.001).


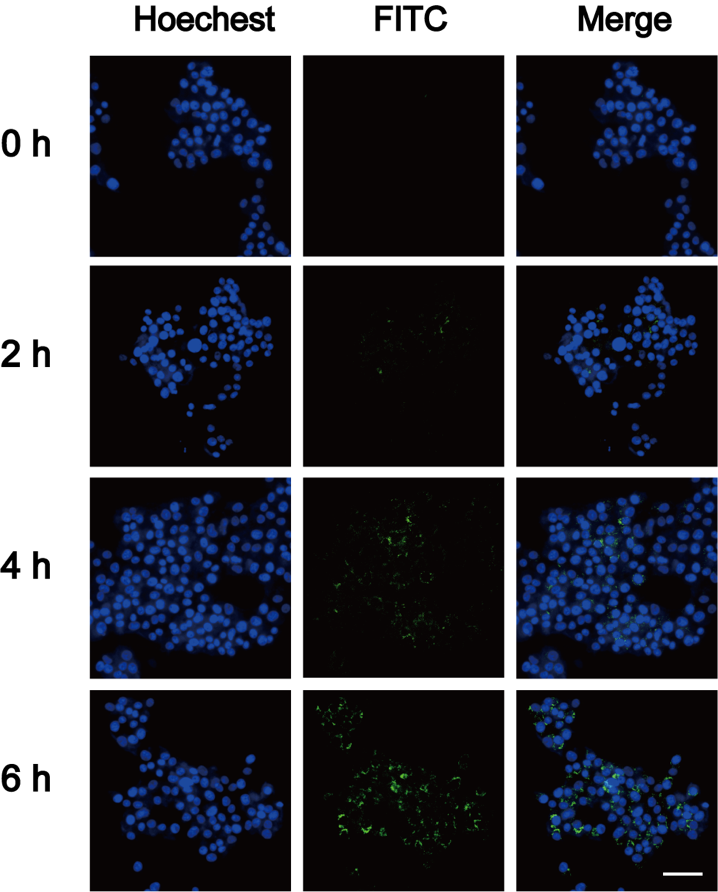


**Figure S6.** Fluorescent microscope imaging of 4',6-diamidino-2-phenylindole (DAPI) stained Cal27 cells incubated with FITC-labeled SAF-SNO NCs for 0, 2, 4, 6 h. Scale bar: 50 µm.


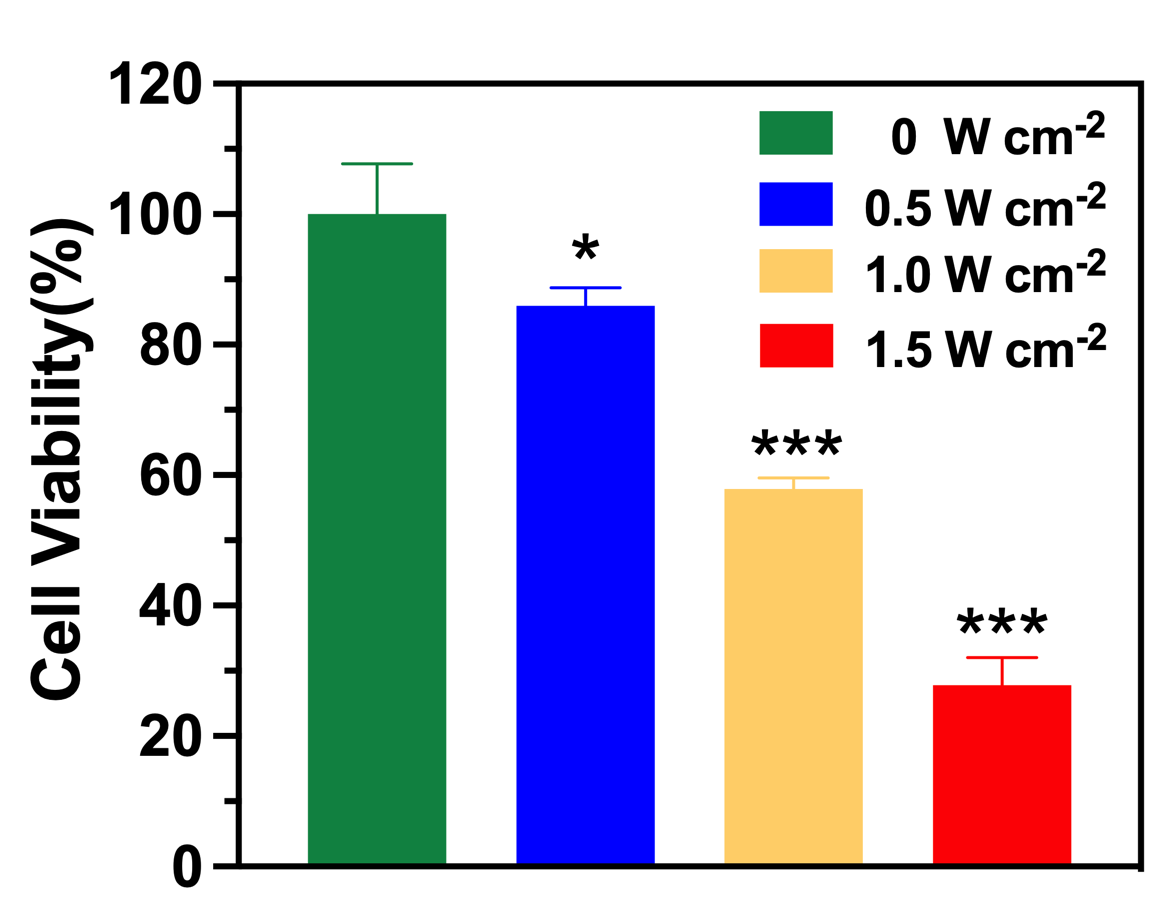


**Figure S7.** Cytotoxicity of SAF–SNO to Cal27 cells under different power densities (0, 0.5, 1.0, and 1.5 W cm^−2^). Statistical significances were calculated *via* Student’s t test, **p*<0.05, ***p*<0.01, ****p*<0.001.


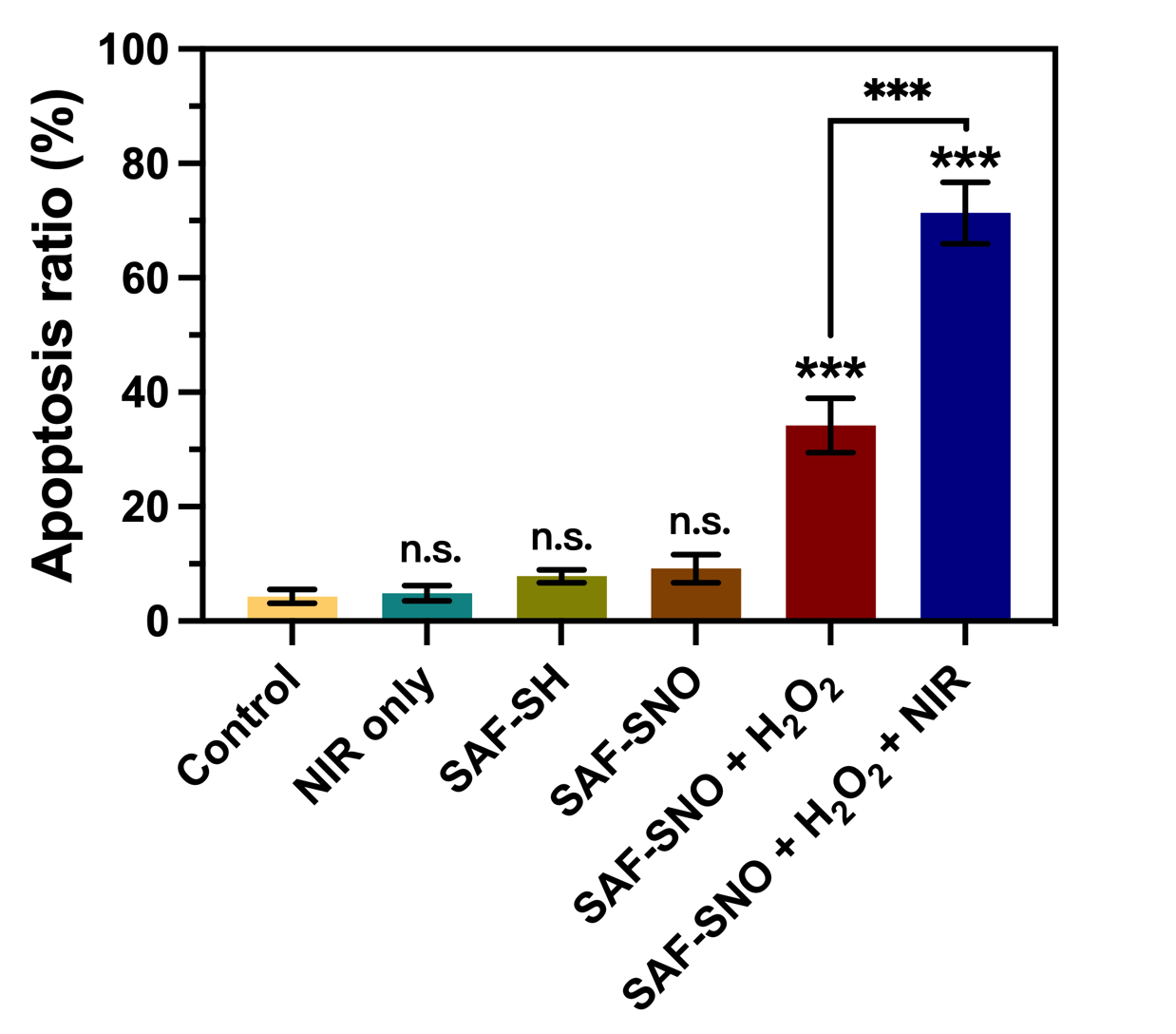


**Figure S8.** The apoptosis ratio of Cal27 cells after different treatments according to the Flow cytometry quantification assay. Statistical significances were calculated *via* Student’s t test, **p*<0.05, ***p*<0.01, ****p*<0.001 and n.s. for non-significant.


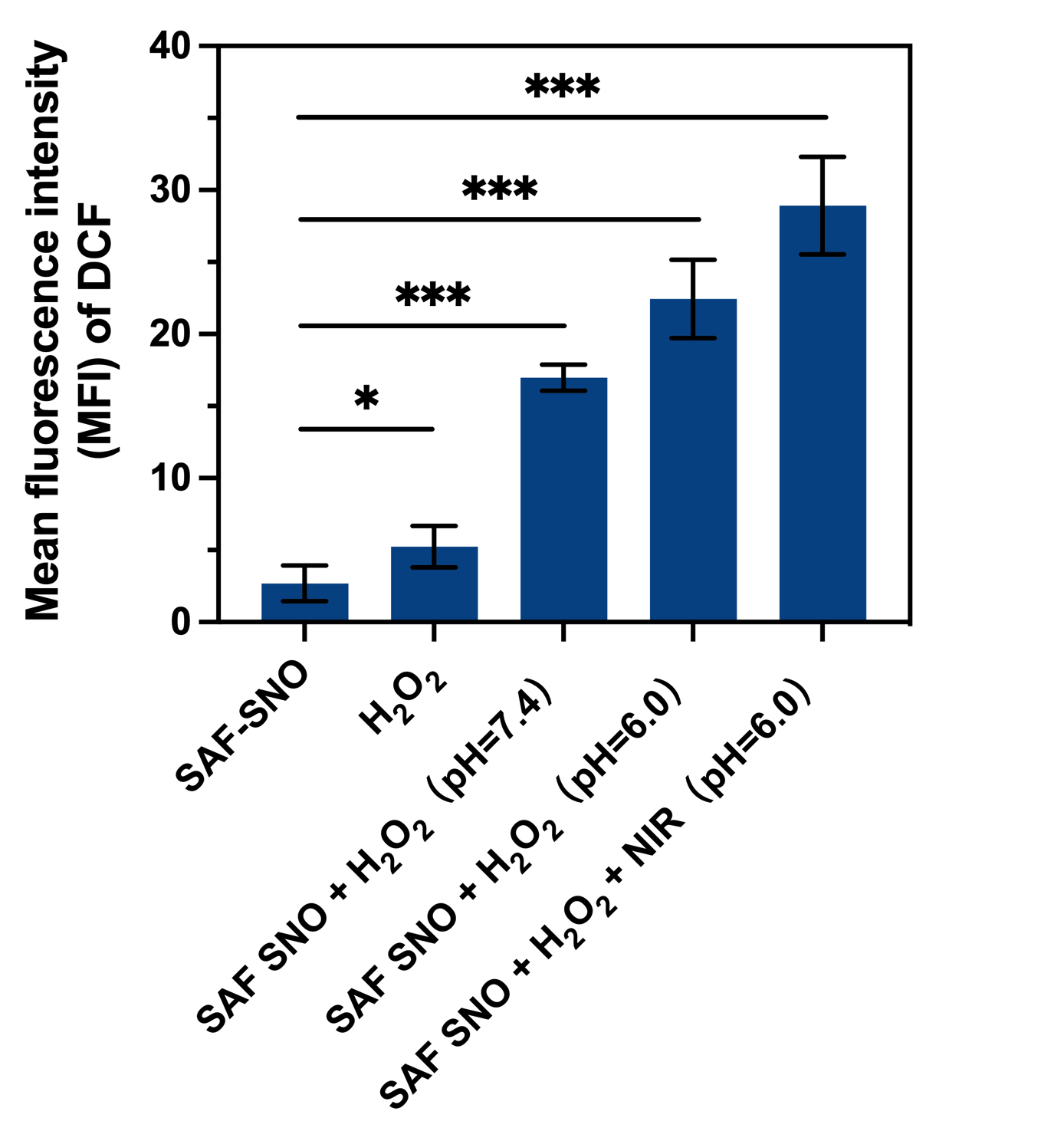


**Figure S9.** Mean fluorescence intensity analysis of CLSM images stained with DCFH-DA after different treatments. Statistical significances were calculated *via* Student’s t test, **p*<0.05, ***p*<0.01, ****p*<0.001 and n.s. for non-significant.


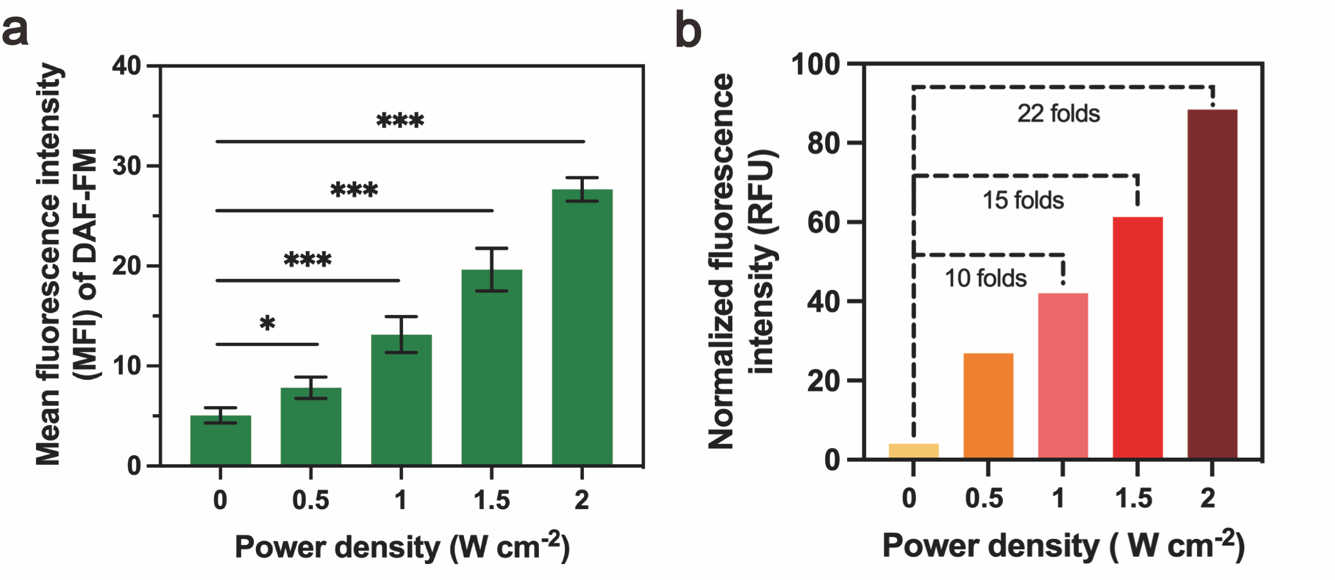


**Figure S10**. (a) Mean fluorescence intensity and (b) Normalized fluorescence intensity analysis of CLSM images stained with DAF-FM DA under different NIR laser power densities (0, 0.5, 1,1.5 and 2 W cm^-2^). Statistical significances were calculated *via* Student’s t test, **p*<0.05, ***p*<0.01, ****p*<0.001 and n.s. for non-significant.


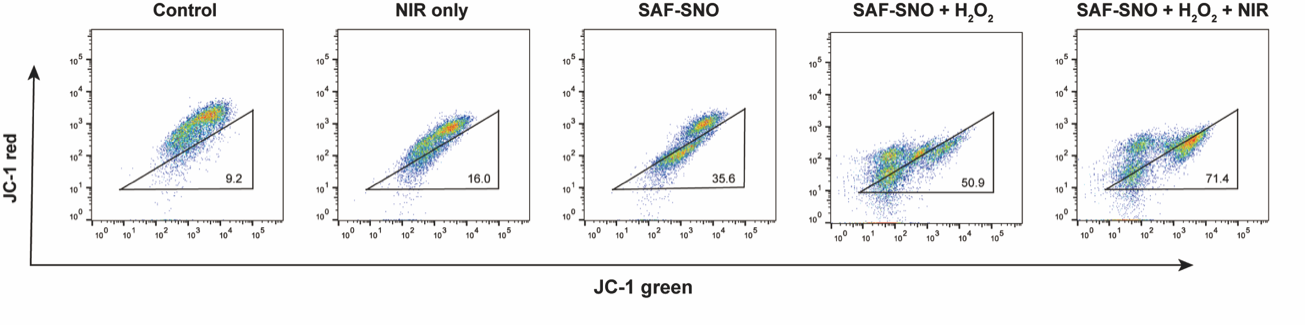


**Figure S11.** Flow cytometry results of JC-1 assay in Cal27 cells were incubated with different treatments: control, NIR only, SAF-SNO, SAF-SNO + H_2_O_2_, SAF-SNO + H_2_O_2_ + NIR.


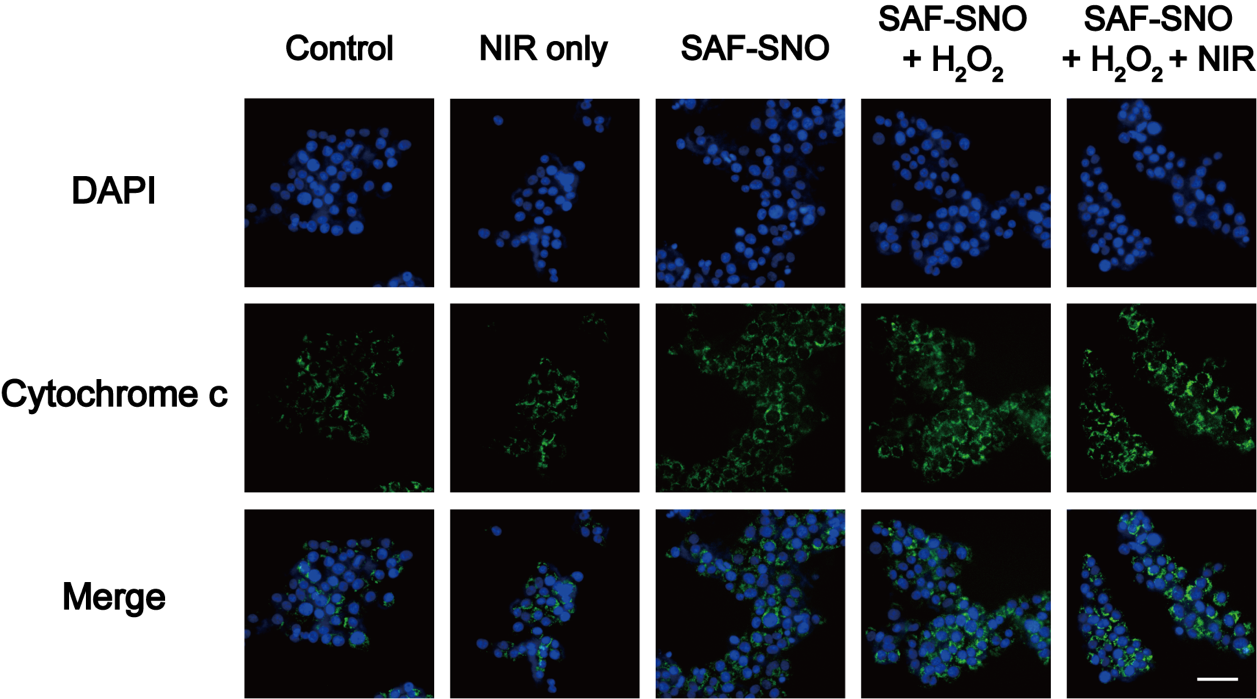


**Figure S12.** Fluorescence images of Cal27 cells stained with Hoechst to identify cell nuclei (blue) and immunofluorescence stained with cytochrome c (green) after receiving different treatments: control, NIR only, SAF-SNO, SAF-SNO + H_2_O_2_, SAF-SNO + H_2_O_2_ +NIR. Scale bar: 50 µm.


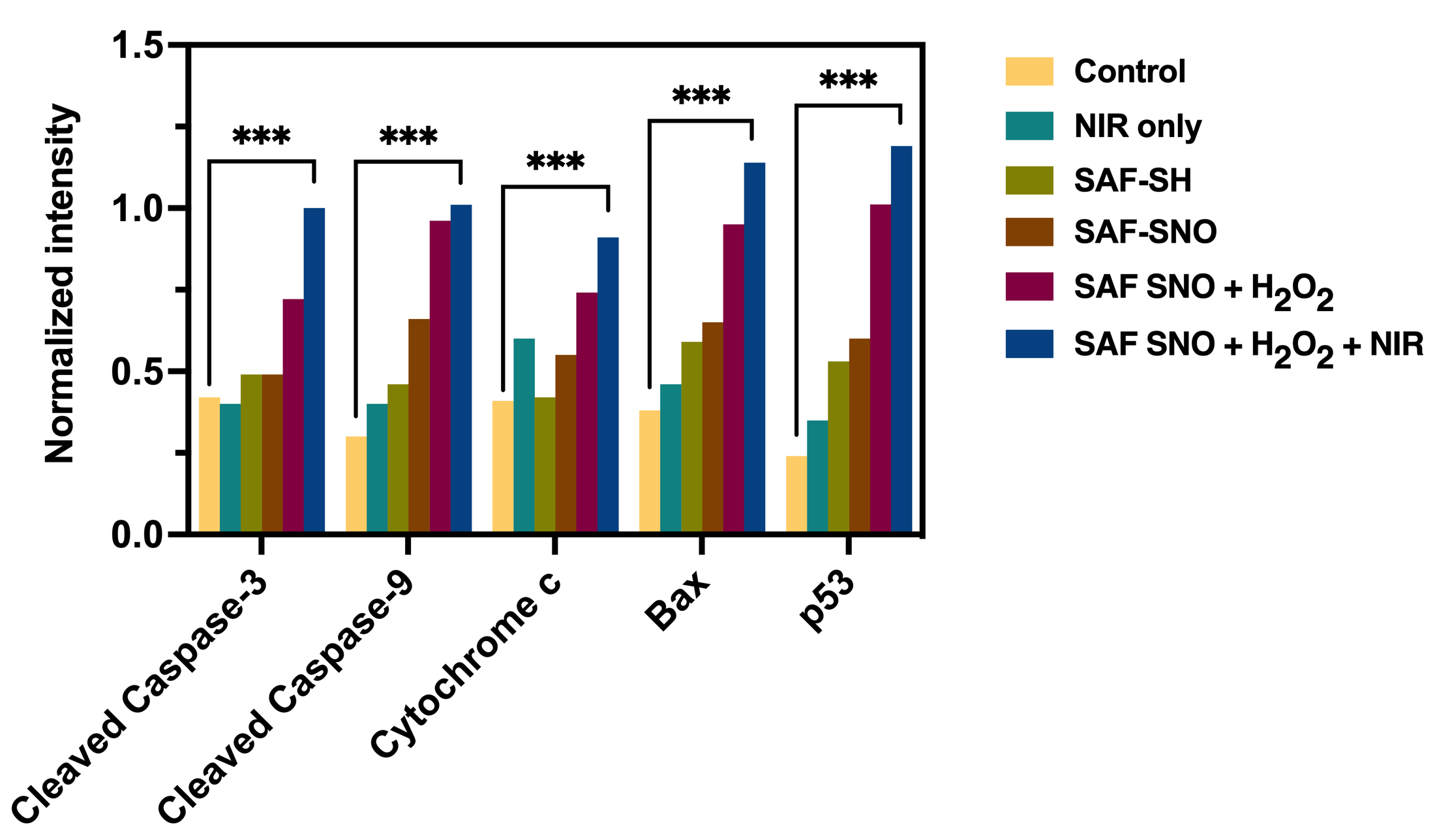


**Figure S13.** Normalized intensity to the β-actin control was quantified for western blot analysis of the p53, Bax, Cytochrome c, cleaved-caspase-3, and cleaved-caspase-9 protein expressions expression in cancer cells subject to different treatments. (*p<0.05, **p<0.01, ***p<0.001).


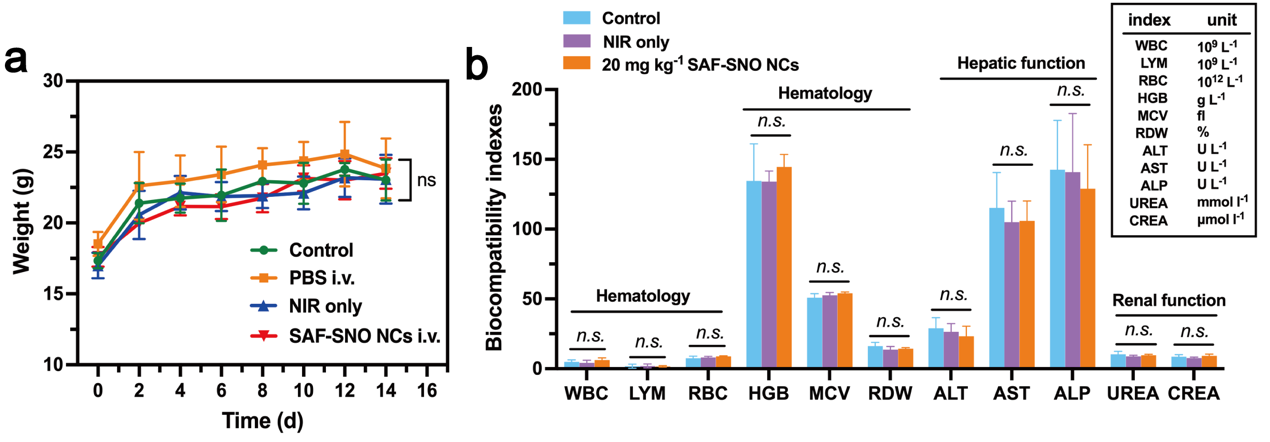


**Figure S14.** (a) Body weights and (b) hematological biochemistry tests of BALB/c nude mice without (saline instead) or with the injection of SAF-SN NCs and SAF-SNO NCs (20 mg kg^-1^) to evaluate the in vivo biocompatibility for 14 d (n = 5, mean ± SD). *P < 0.05, n.s. (non-significant).


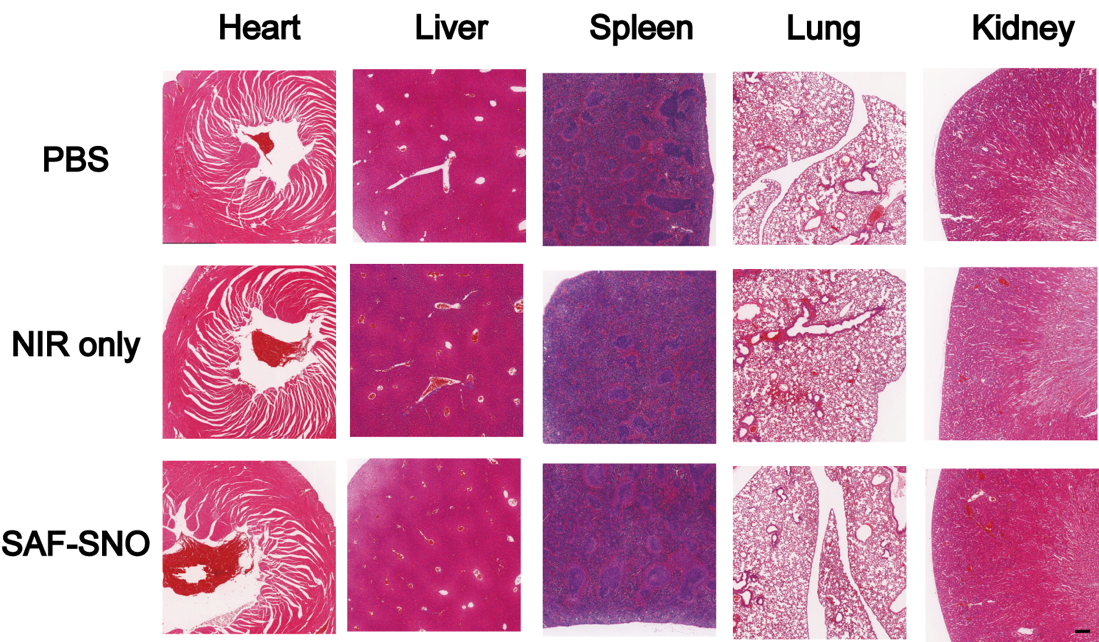


**Figure S15.** H&E staining major organs (heart, liver, spleen, lung and kidney) dissected from BALB/c nude mice without (saline instead) or with the injection of SAF-SN NCs and SAF-SNO NCs (20 mg kg^-1^) to evaluate the in vivo biocompatibility after 14 d. Scale bar: 100 μm.


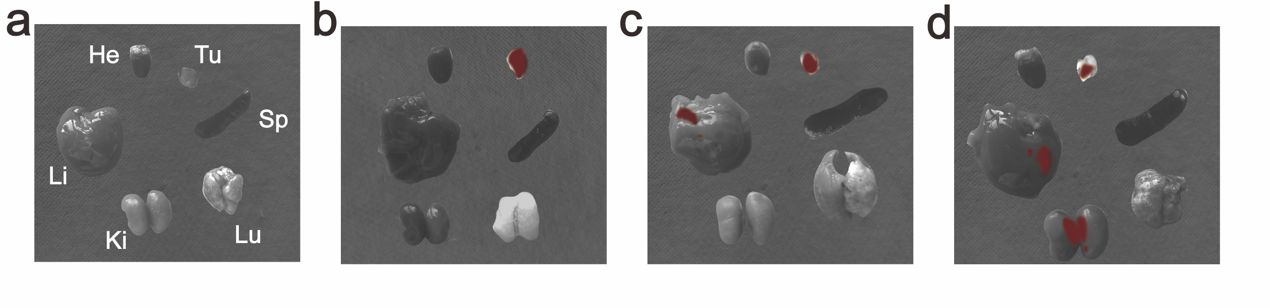


**Figure S16.** In vivo fluorescence images of dissected major organs and tumors from cal27-tumor-bearing nude mice at varied time points (a)Pre-, (b)Post-, (c)4 h, and (d)24 h after once intratumoral injection with Cy5.5-labeled SAF-SNO NCs.


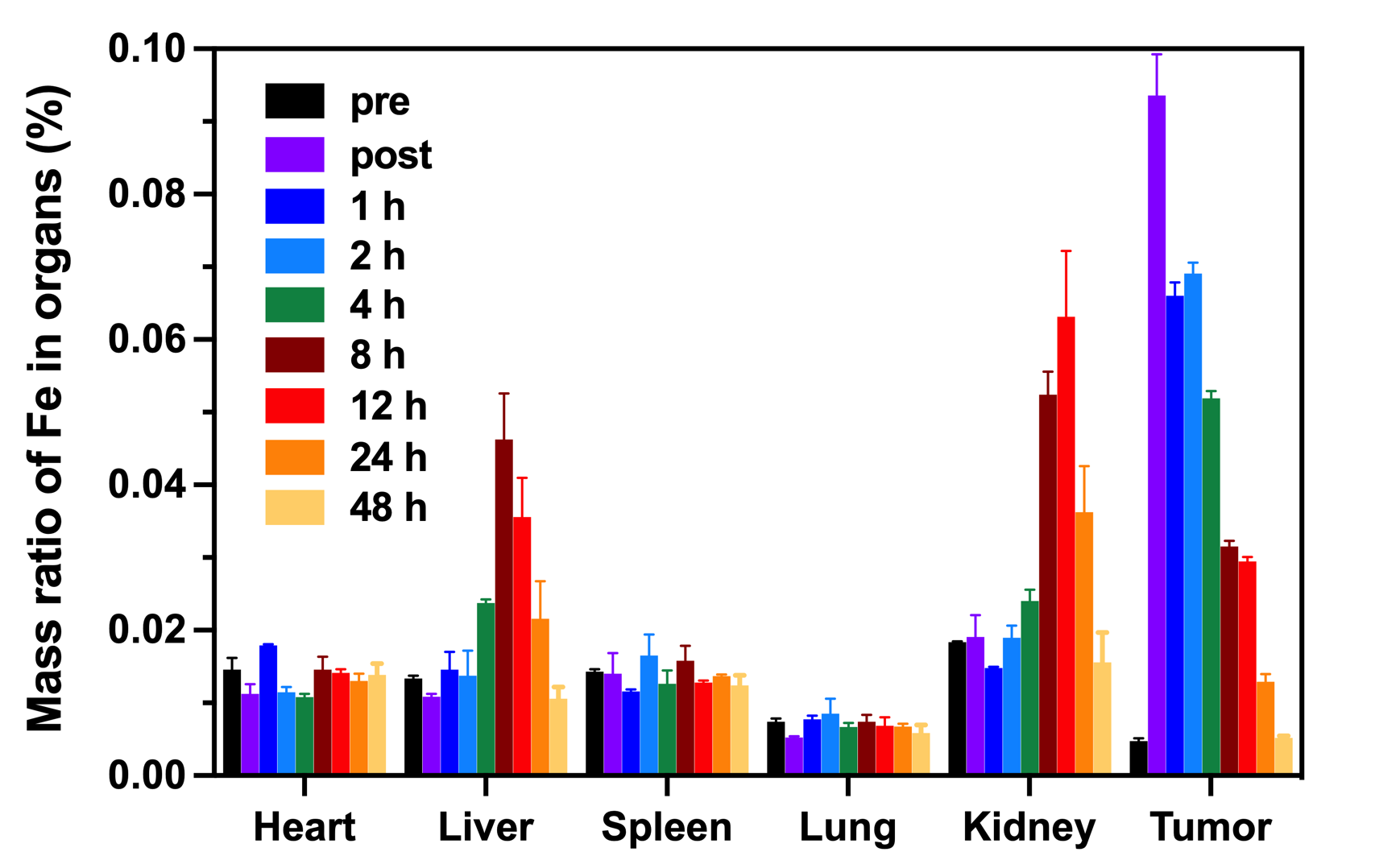


**Figure S17.** In vivo biodistribution of Fe (% of Fe per gram of tissues) in tumors and major organs measured by ICP-MS in cal27-tumor-bearing nude mice (n = 3) at varied time points (Pre-, Post-, 1 h, 2 h, 4 h, 8 h, 12 h, 24 h and 48 h) after once intratumoral injection with SAF-SNO NCs.


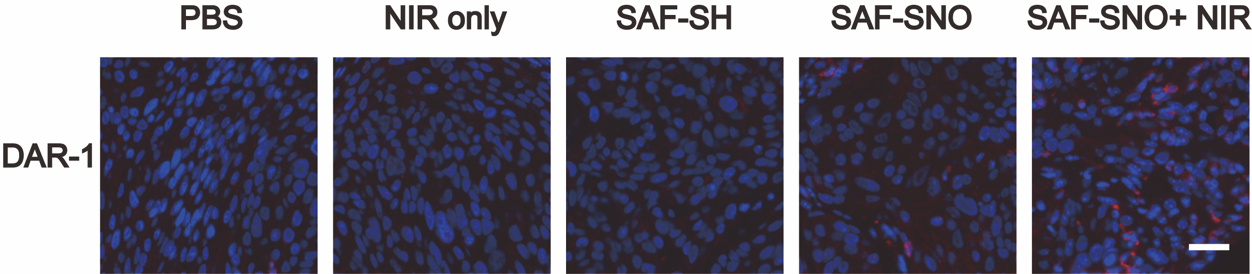


**Figure S18**. Immunofluorescence staining of the distribution of NO released from isolated tumors by DAR-1 fluorescence probe (red) after different treatment groups, including control, NIR only, SAF-SH NCs i.t. (10 mg kg^−1^), SAF-SNO NCs i.t. (10 mg kg^−1^) and SAF-SNO NCs i.t. (10 mg kg^−1^) + NIR laser irradiation for 5 min. Scale bars: 25 μm.


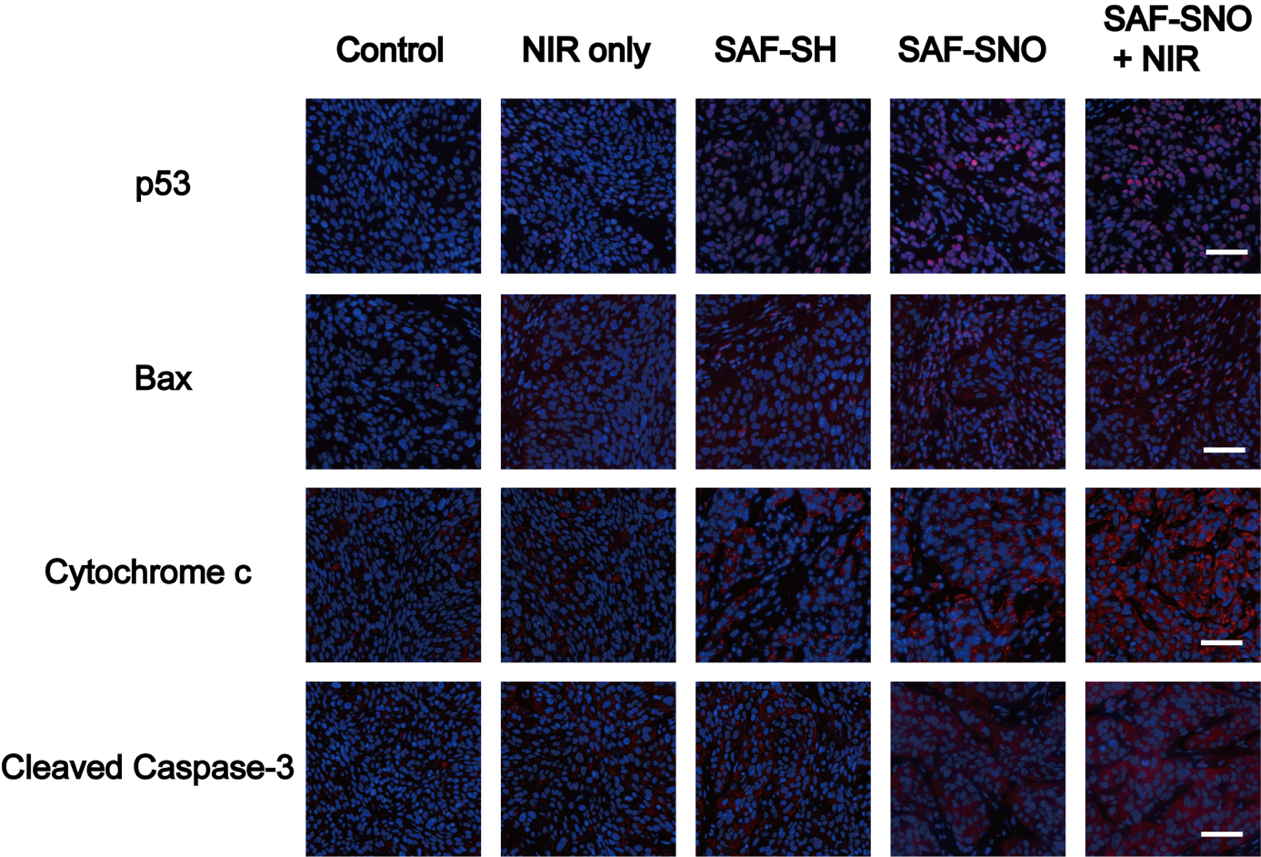


**Figure S19**. Immunofluorescence staining of the apoptosis-relative marker of p53, Bax, Cytochrome c, cleaved Caspase-3 to evaluate their expression in the isolated tumors from different treatment groups, including control, 808 nm laser irradiation only, SAF-SH NCs i.t. (10 mg kg^−1^), SAF-SNO NCs i.t. (10 mg kg^−1^) and SAF-SNO NCs i.t. (10 mg kg^−1^) + NIR laser irradiation for 5 min. Scale bars: 50 μm.
